# Mechanism of secondary recrystallization of Goss grains in grain-oriented electrical steel

**DOI:** 10.1080/14686996.2017.1341277

**Published:** 2017-07-14

**Authors:** Yasuyuki Hayakawa

**Affiliations:** ^a^ Steel Research Laboratory, JFE Steel Corporation, Kurashiki, Japan

**Keywords:** Grain-oriented electrical steel, secondary recrystallization, primary recrystallization, texture, grain growth, inhibitor, 40 Optical, magnetic and electronic device materials, 106 Metallic materials, 206 Energy conversion / transport / storage / recovery

## Abstract

Since its invention by Goss in 1934, grain-oriented (GO) electrical steel has been widely used as a core material in transformers. GO exhibits a grain size of over several millimeters attained by secondary recrystallization during high-temperature final batch annealing. In addition to the unusually large grain size, the crystal direction in the rolling direction is aligned with <001>, which is the easy magnetization axis of α-iron. Secondary recrystallization is the phenomenon in which a certain very small number of {110}<001> (Goss) grains grow selectively (about one in 10^6^ primary grains) at the expense of many other primary recrystallized grains. The question of why the Goss orientation is exclusively selected during secondary recrystallization has long been a main research subject in this field. The general criterion for secondary recrystallization is a small and uniform primary grain size, which is achieved through the inhibition of normal grain growth by fine precipitates called inhibitors. This paper describes several conceivable mechanisms of secondary recrystallization of Goss grains mainly based on the selective growth model.

## Graphical abstract

**Figure:**
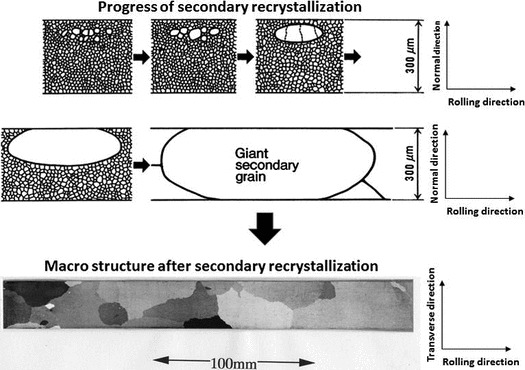

## 1. Introduction

Since the beginning of the 21st century, saving electrical energy has become increasingly crucial from the viewpoints of reducing greenhouse gas emissions and conserving limited natural resources. The steel industry is often regarded as a major energy consumer; however, the use of electrical steel also saves energy. Besides, electrical steel dominates the market in the field of soft magnetic materials.

Electrical steel is divided into two types, namely, grain-oriented (GO) steel and non-oriented (NO) steel. GO steel is mainly used as a core material for transformers, while NO steel is used in the cores of electric motors. Both types of electrical steel are indispensable for energy transportation and conversion.

The most important factor in electrical steel is the Si content. In 1900, Barret, Hegar and Hadfield [1] discovered that iron loss can be reduced by the addition of Si. Si increases electrical resistivity and hence reduces the eddy current loss that is generated in electromagnetic induction.

Production of both types of electrical steel has increased steadily in order to meet the needs of global economic growth. The fundamental aim in the development of electrical steel is improvement of magnetic properties to reduce energy loss in the iron core. The focus of this paper is the technical background of GO steel production.

**Figure 1.**
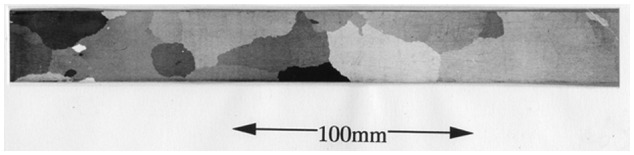
Macrostructure of a typical HGO steel sheet (reproduced with permission from [3] © 1998 The Iron and Steel Institute of Japan).

**Figure 2.**
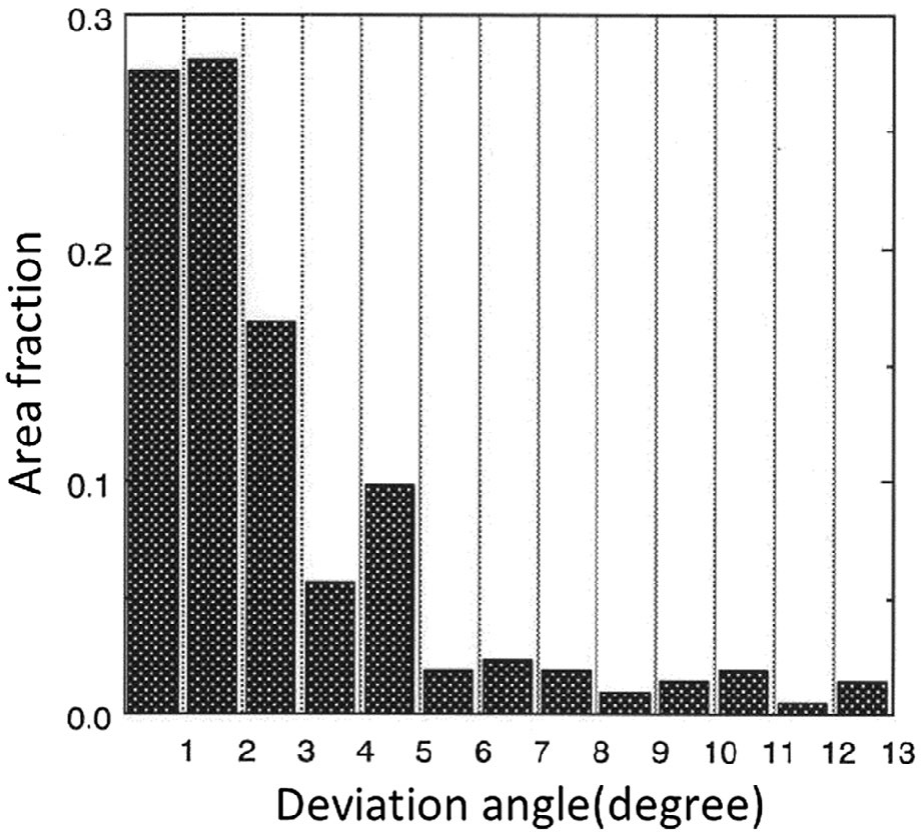
Distribution of deviation angle from {110}<001> orientation in secondary recrystallization texture (reproduced with permission from [3] © 1998 The Iron and Steel Institute of Japan).

GO steel sheets usually have a thickness of between 0.23 mm and 0.35 mm and a large grain size, ranging from approximately 5 mm to 50 mm, which is much larger than the thickness of the sheet. An example of the macrostructure of a GO steel sheet is shown in Figure 1. An insulation coating is applied to both surfaces of the sheet to avoid eddy currents between the stacked sheets that comprise a transformer core. In addition to the unusually large grain size, the most characteristic feature of the base metal is the fact that the crystal direction in the rolling direction is highly oriented to <001>. As discovered by Honda and Kaya [2] in 1926, <001> in α-iron is the easy magnetization axis. An example of the distribution of the deviation angles from {110}<001> in highly grain-oriented (HGO) steel is shown in Figure 2 [3]. As can be understood from Figure 2, the deviation angle from {110}<001> is mostly reduced to less than 5°. Considering the fact that the frequency of {110}<001> with deviation angles under 5° is less than 0.001 in a random texture, the sharpness of the texture of this material is truly remarkable.

**Figure 3.**
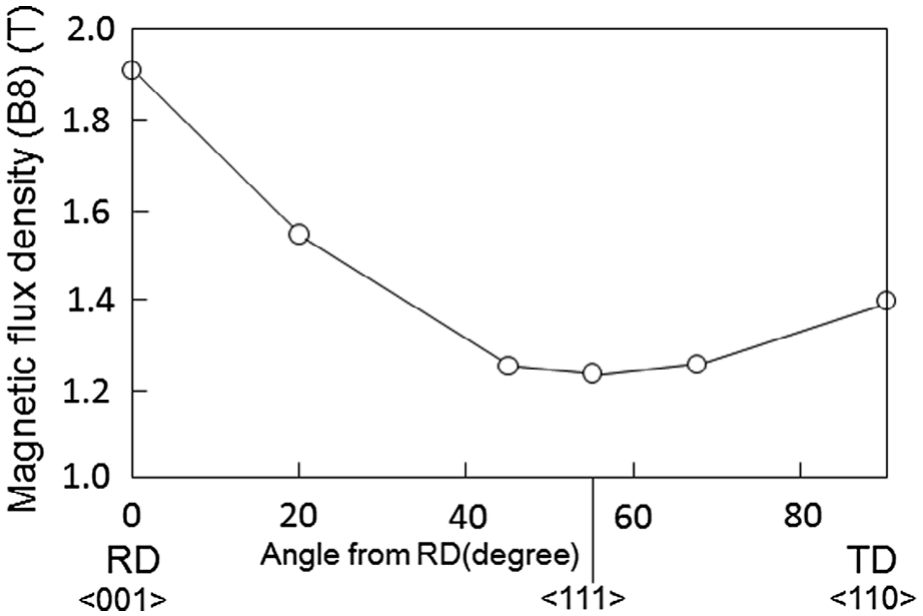
Magnetic flux density (B8) of HGO steel along various crystallographic directions [4]. RD stands for rolling direction and TD for transverse direction.

The magnetic flux density of GO steel with a magnetization force of 800 A/m (B8) in various directions is shown in Figure 3 [4]. As shown in this figure, magnetic flux density (B8) displays its maximum value in the rolling direction (RD) corresponding to <001> and its minimum value in the direction corresponding to <111>.

In 1934, Goss [5] invented GO steel, which was produced through a two-stage cold rolling process with intermediate annealing between the cold rolling stages. Subsequent research proved that the crystal orientation produced by Goss’s invention corresponded to {110}<001> [6]. Today, {110}<001> is called the ‘Goss orientation’, and, interestingly, ‘Goss’ can also be used as an abbreviation for grain-oriented silicon steel, as pointed out by Matsuo [7].

**Figure 4.**
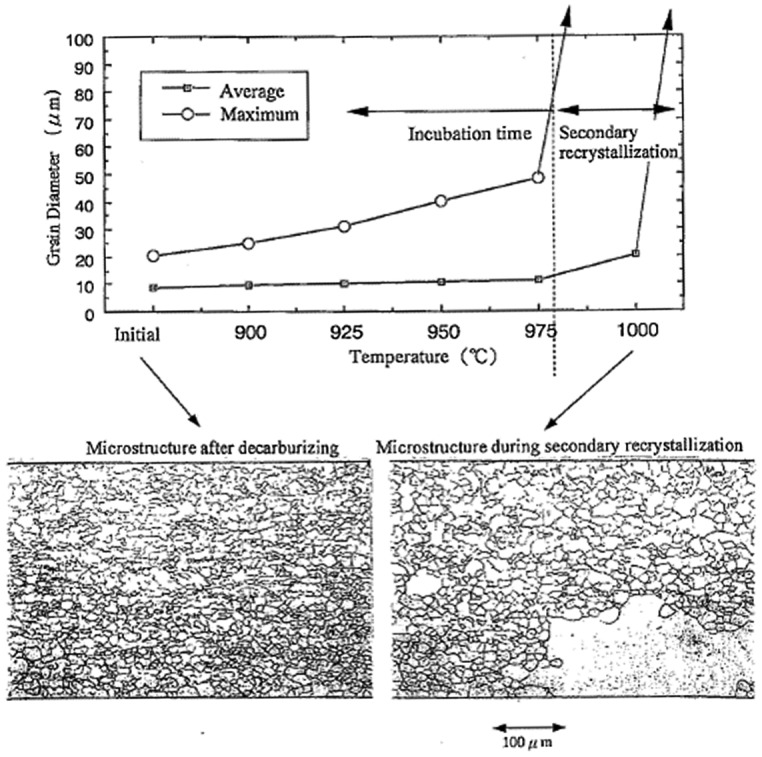
Top: grain growth as a function of temperature (°C). Bottom: micrographs of longitudinal cross-sections (reproduced with permission from [3] © 1998 The Iron and Steel Institute of Japan).

The most important and unique point of the GO manufacturing process is the final high-temperature batch annealing process. Secondary recrystallization of Goss grains is achieved in the course of this process. The grain growth behavior of HGO steel in final batch annealing is shown in Figure 4 [3]. Secondary recrystallization is the phenomenon in which a certain very small number of Goss grains grow selectively (about one in 10^6^ primary grains) at the expense of many other primary recrystallized grains. The development history of GO steel production has been reviewed by Xia et al. [8].

## 2. Mechanism of secondary recrystallization of Goss grains

The question of why the Goss orientation is exclusively selected during secondary recrystallization has long been a main research subject in this field. Some of the hypotheses are introduced in this section.

### 2.1. Role of inhibitors

The general criterion for secondary recrystallization is a small and uniform primary grain structure, which is achieved through the inhibition of normal grain growth by fine precipitates or solute atoms and/or through a strong primary recrystallization texture. In other words, impurity inhibition and/or texture inhibition are involved.

**Figure 5.**
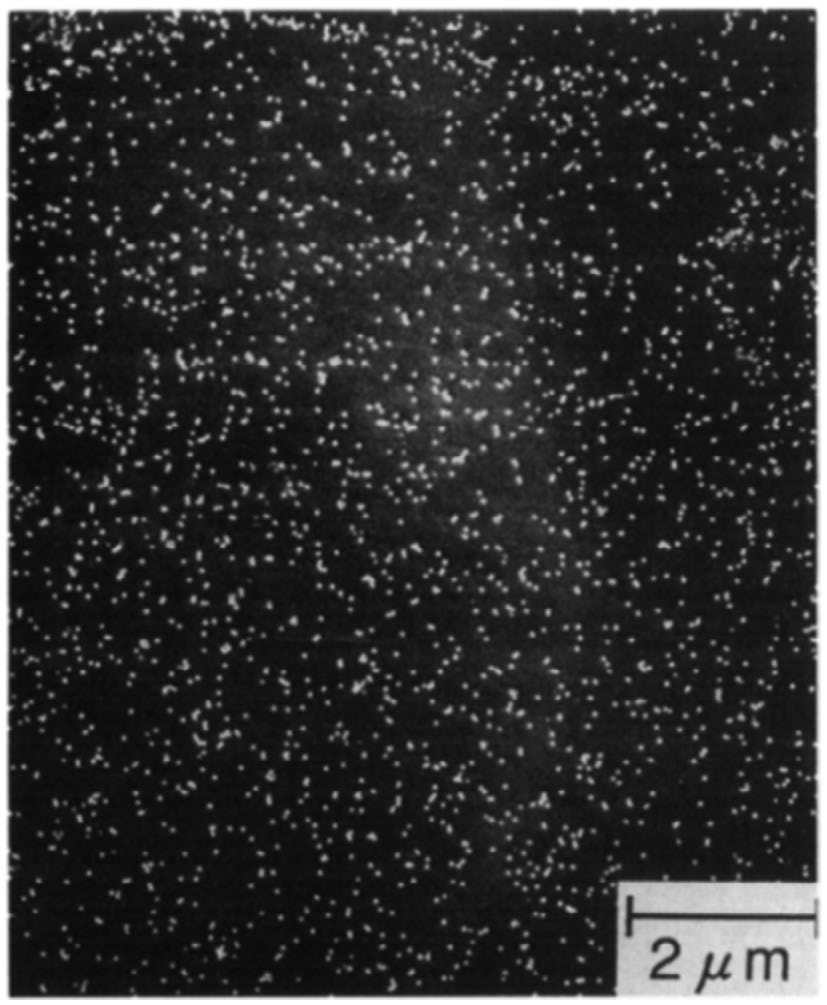
Inhibitors observed by SEM (reproduced with permission from [10] © 1993 Springer).

In 1958, May and Turnbull [9] clarified the necessity of dispersed precipitates of MnS for the development of secondary recrystallization. They suggested a function whereby the precipitates inhibit normal grain growth and thus maintain the driving force for secondary recrystallization. Precipitates that inhibit normal grain growth are now called ‘inhibitors’. An example of the MnSe inhibitor distribution observed by scanning electron microscopy (SEM) is shown in Figure 5 [10].

Swift [11] related the diffusion controlled coarsening of MnS precipitates to the onset of secondary recrystallization. Markuszewicz et al. [12] investigated the relation between the stability of inhibitors and the formation of secondary recrystallization. At a high content of inhibitors, especially that of stable inhibitors such as Al_2_O_3_, the growth of all grains was restrained uniformly, and secondary recrystallization did not occur. A favorable ratio of inhibitors which easily undergo decomposition resulted in the selective growth of Goss grains.

**Figure 6.**
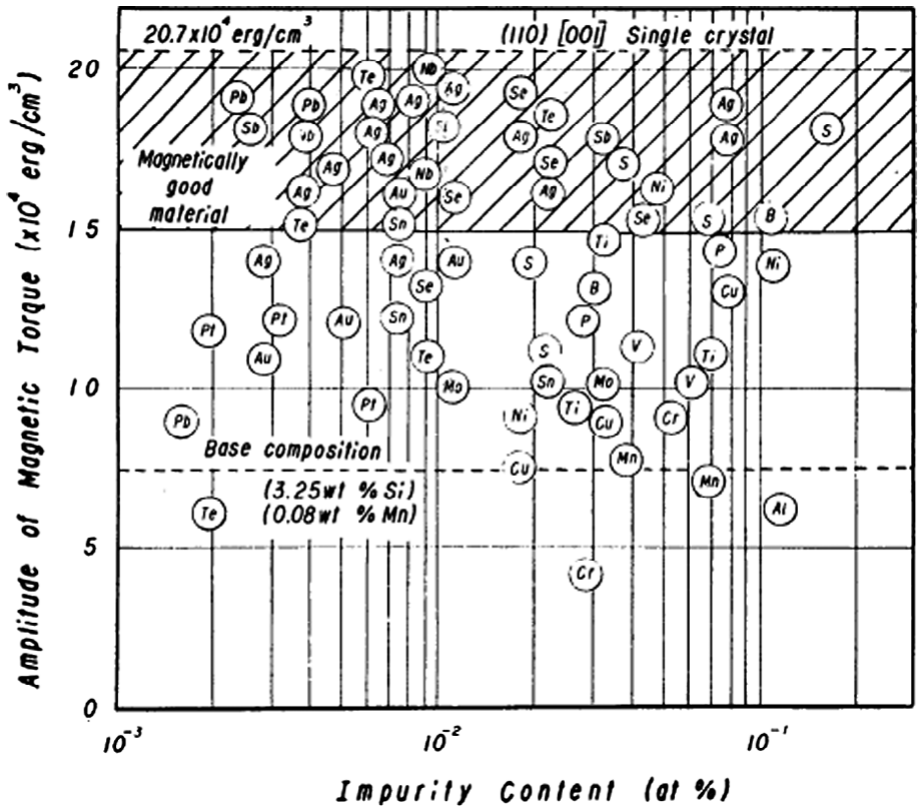
Effect of impurity content on amplitudes of magnetic torque of 3.25 wt% Si-steel strip annealed at 1100 °C (reproduced with permission from [18] © 1963 The Japan Institute of Metals and Materials).

Following the above-mentioned suggestion by May and Turnbull [9], many experiments were performed in a search for new kinds of inhibitors, including sulfides, nitrides, carbides and/or solute elements. Reported examples of such inhibitors include VN by Fiedler [13], AlN by Taguchi et al. [14], TiC, VN and NbC by Matsuoka [15] and MnSe by Fiedler [16]. Saito [17,18] carried out an extensive investigation of the effect of solute elements and found that the addition of Pb, Sb, Nb, Ag, Te, Se and S was effective for improving magnetic property, as shown in Figure 6, and that these inhibitors also improved magnetic properties by promoting a higher accumulation toward the Goss orientation in the secondary recrystallization texture.

Grenoble and Fiedler [19] showed that sulfur and nitrogen, when present together as solutes, enable secondary recrystallization to occur. Grenoble [20] also reported secondary recrystallization with a combination of solute S, N and B. Use of a combination of inhibitors and solute elements contributed to the stability of secondary recrystallization and improved magnetic properties.

### 2.2. Nucleation and growth

Oriented nucleation and selective growth are frequently discussed as analogies to recrystallization. However, unlike recrystallization, the definition of nucleation in secondary recrystallization is rather vague. The possibility of abnormal grain growth to over two times the average size has been suggested based on the normal grain growth model proposed by Hillert [21]. However, even assuming the existence of grains with larger-than-average sizes, it is not clear whether these grains eventually become secondary recrystallized grains. The extreme rareness of nucleation is an essential difficulty, in that it has been estimated that only one of 10^6^ primary grains will become a successful Goss grain as a nucleus for secondary recrystallization. This implies that one must investigate at least 10^6^ grains in order to observe one nucleus.

Leaving aside the issue of the exact definition, the results of orientation measurements have revealed that growing grains which are several times larger than the average grain size display the Goss orientation. Thus, if these grains are defined here as nuclei, the concept of oriented nucleation is applicable.

### 2.3. Observation of nucleation process

**Figure 7.**
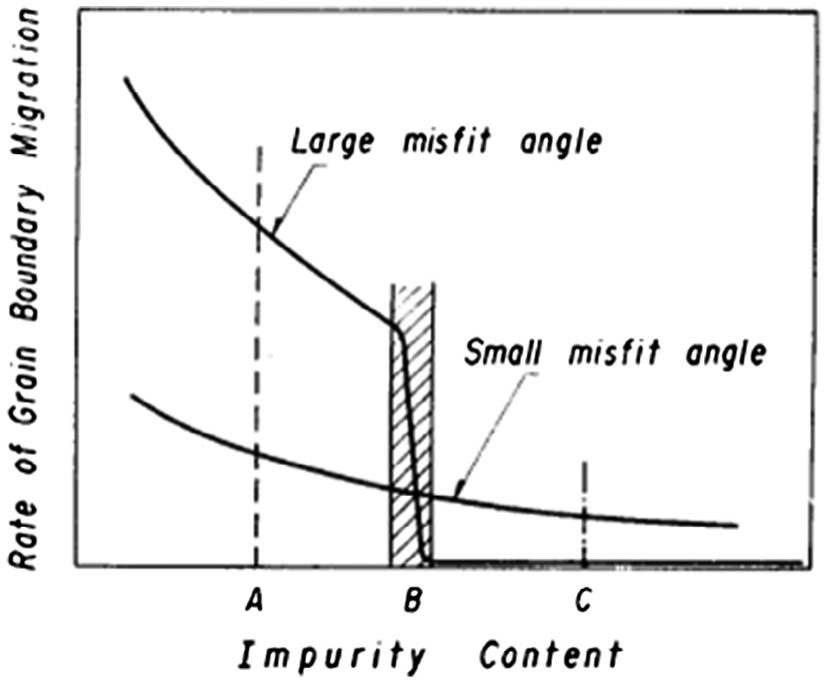
Schematic of relation between rate of grain boundary migration and impurity content for large and small misfit angles (reproduced with permission from [22] © 1970 The Japan Institute of Metals and Materials).

**Figure 8.**
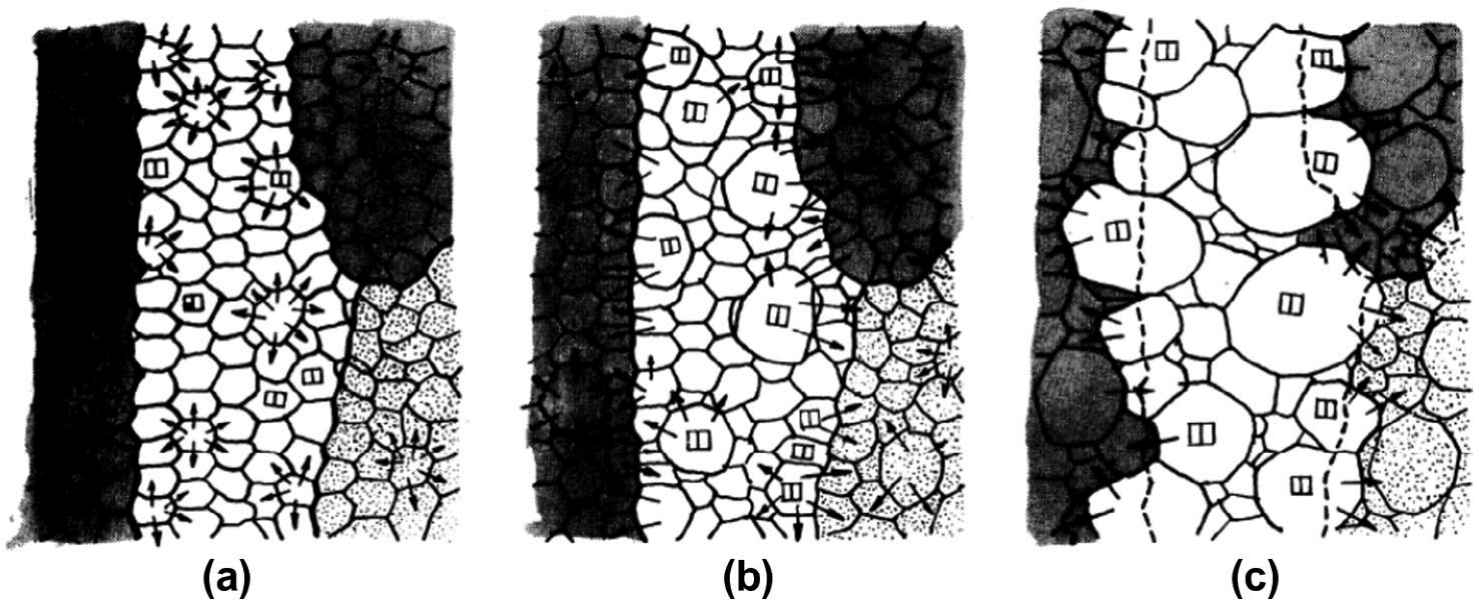
Schematics of abnormal grain growth of Goss-oriented grains. (a) Primary recrystallization structure. (b) Growth of grains having mutually close orientations. (c) Abrupt growth of Goss grains into other colonies of recrystallized grains, above dissociation temperature of sulfide (reproduced with permission from [23] © 1971 The Japan Institute of Metals and Materials).

Nakae and Tagashira [22,23] investigated the growth of Goss grains in a Fe-3%Si alloy containing sulfur. A schematic of the relation between mobility and the impurity content which they proposed is shown in Figure 7. Mobility rises sharply for high-angle grain boundaries under certain impurity content, whereas the mobility rise is gradual for low-angle grain boundaries. These changes cause a reversal of the mobility of high- and low-angle grain boundaries, depending on the impurity content level. The process of secondary recrystallization is shown in Figure 8. Observations by Nakae and Tagashira [22,23] provide insight into the meaning of incubation time, namely, the division of the nucleation and growth stages.

Formation of secondary recrystallization nuclei in the subsurface layer has been reported by many researchers. In the case of HGO steel using AlN and MnS as inhibitors with single-stage cold rolling, Sakai et al. [24] investigated nucleation behavior by micro-facet etch pit figures. They found that colonies of Goss grains were situated in the subsurface layer and that the colonies of Goss grains in the vicinity of the steel surface showed selective growth during subsequent secondary recrystallization annealing.

**Figure 9.**
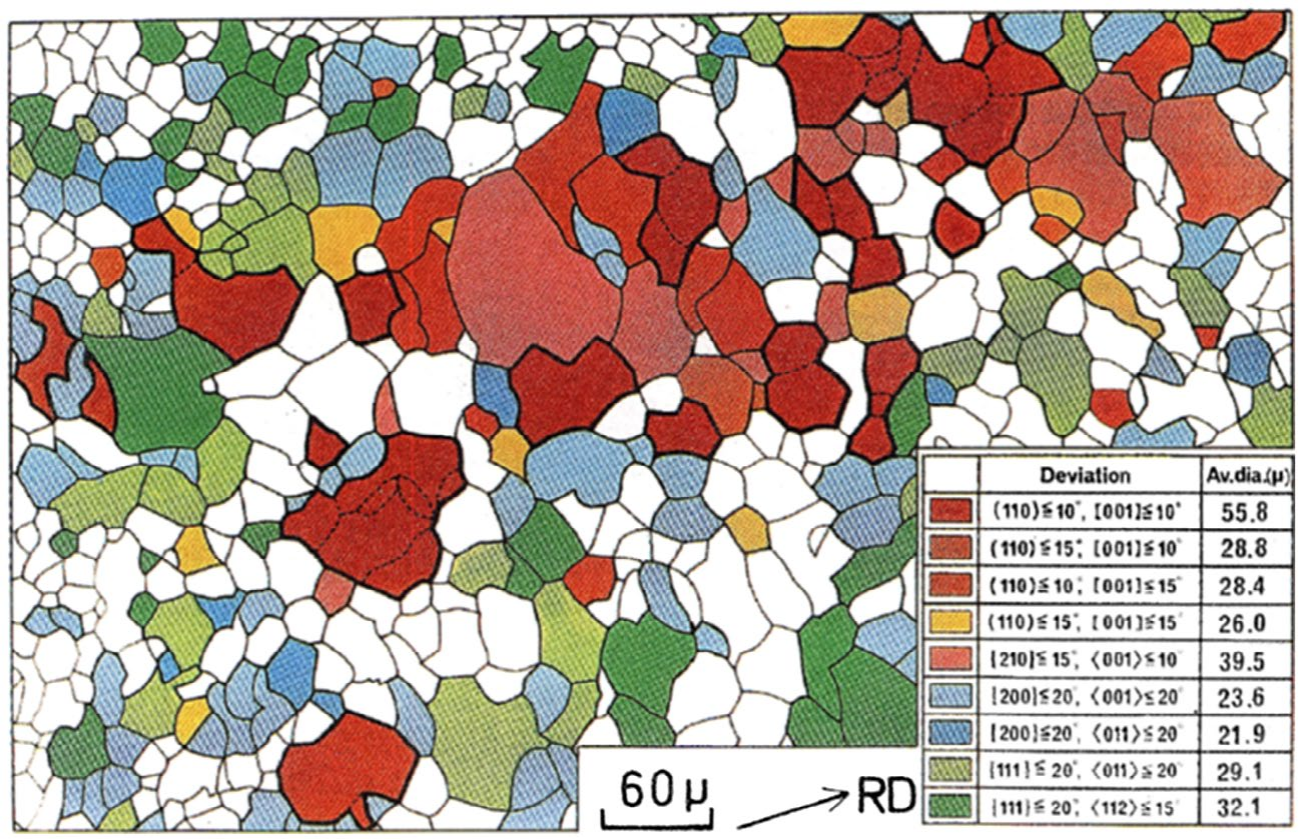
Orientations of primary recrystallized grains at 1/10 depth under surface (reproduced with permission from [25] © 1984 The Iron and Steel Institute of Japan).

**Figure 10.**
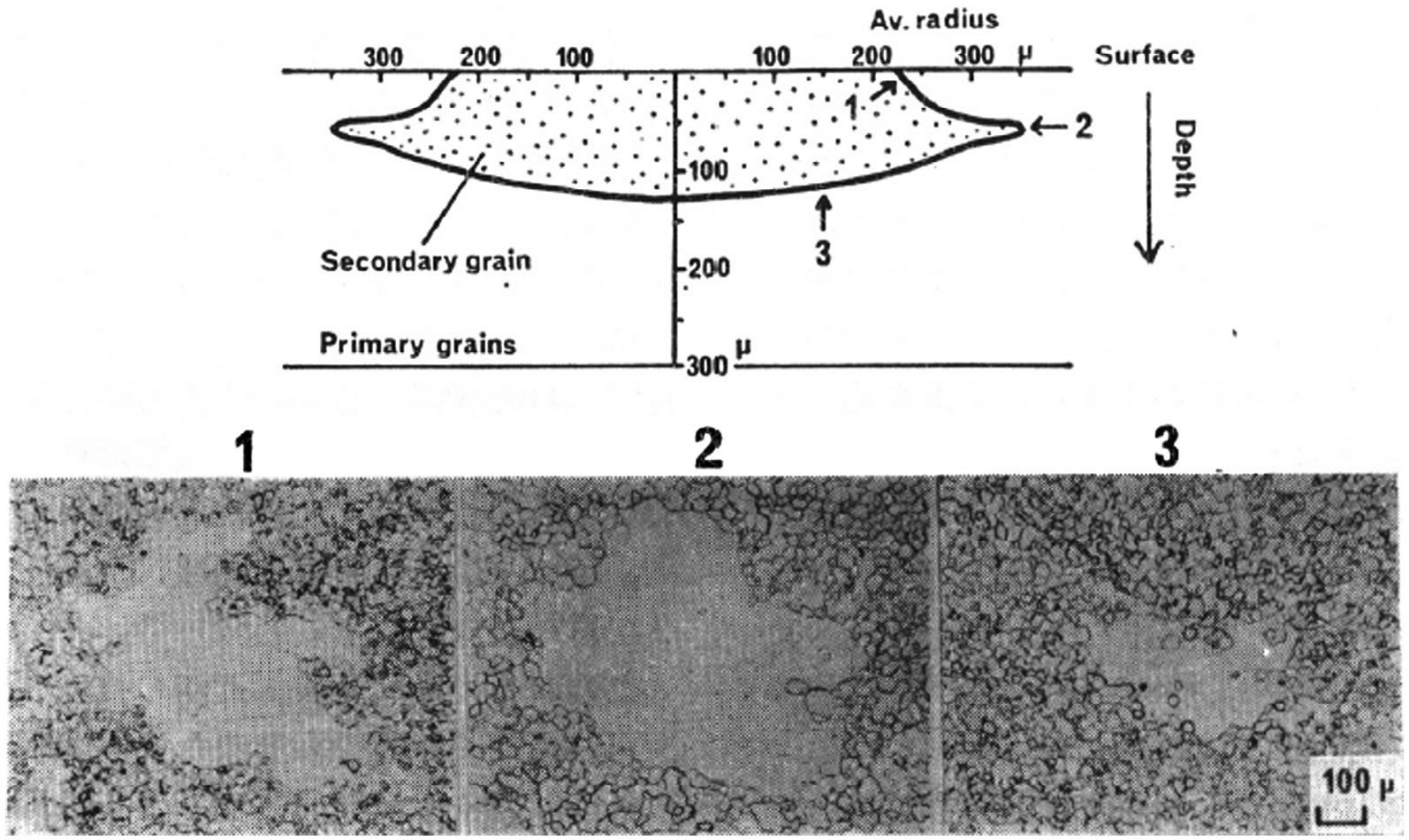
Cross-sectional morphology of a growing secondary grain. Micrographs 1, 2 and 3 at the bottom show the secondary grain at depths of about 15, 40 and 140 μm, respectively, under the surface of the steel sheet (reproduced with permission from [25] © 1984 The Iron and Steel Institute of Japan).

In a study of HGO steel using MnSe and Sb as inhibitors with two-stage cold rolling, Inokuti [25,26] investigated the primary recrystallization texture and the texture after subsequent annealing at 850 °C by transmission Kossel (TK) examination. As shown in Figure 9, Goss grains formed colonies at the 1/10 of sample thickness beneath the surface of the primary recrystallized sheet. Selective growth of Goss grains took place in a zone at the 1/10 of sample thickness beneath the surface of the primary recrystallized steel sheet, as shown in Figure 10.

**Figure 11.**
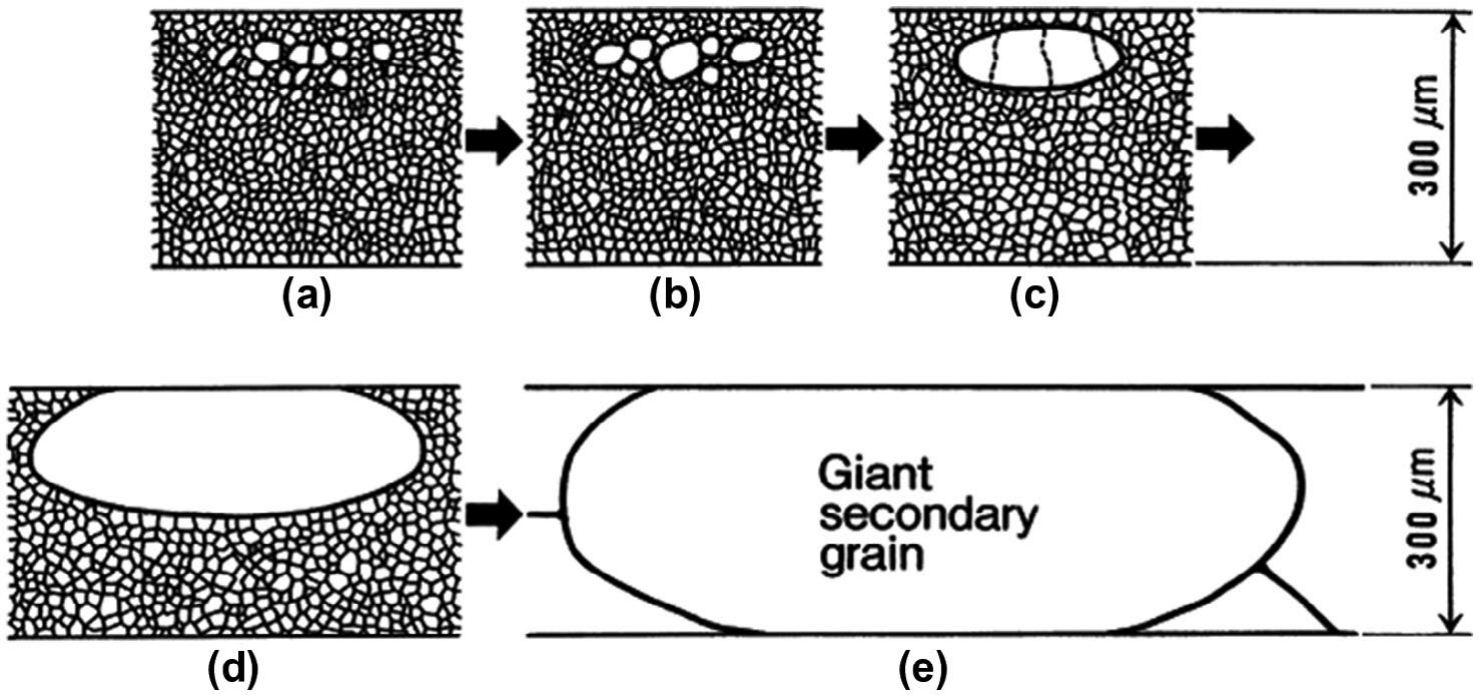
Schematic of cross-section of steel sheet showing nucleation and preferential growth of secondary recrystallized Goss grains in GO steel sheet [26].

A schematic of the nucleation and growth stages is shown in Figure 11.

In spite of the very different recrystallization textures of HGO steel produced by the single-stage and the two-stage cold rolling processes, it is worth noting that the Goss nuclei formed in the subsurface layer. In both cases, the Goss nuclei formed with a pancake morphology having a diameter of several 100 μm in the rolling plane and a thickness of 20 μm.

### 2.4. Growth rate

**Figure 12.**
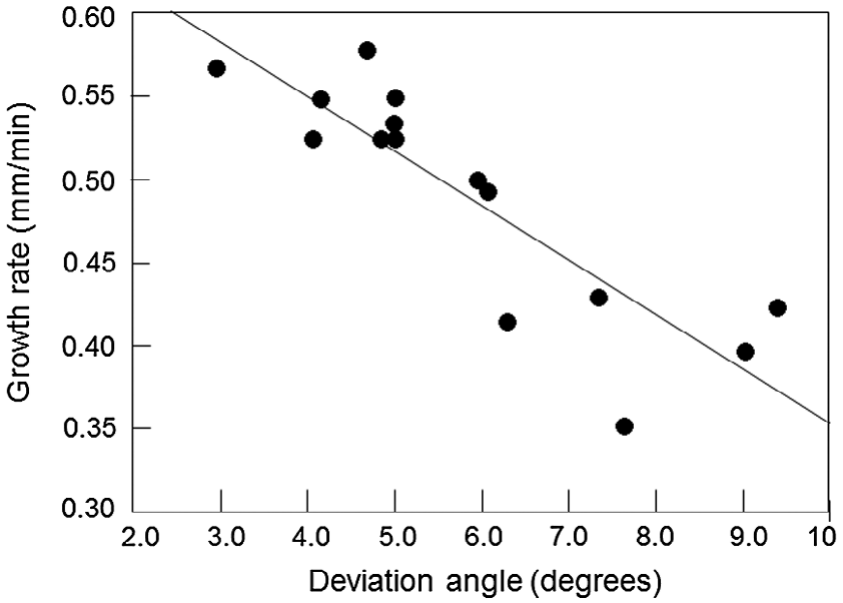
Relation between deviation angle from ideal Goss orientation and growth rate of secondary recrystallized grains [29].

The experimental data on the growth rate of secondary recrystallized grains were dependent on the orientations of the secondary recrystallized grains. Grains having the ideal Goss orientation usually have an advantage in growth over deviated grains [27–29]. The relationship between the growth rate and the deviation angle [29] is shown in Figure 12.

Matsuo [7] reported that Goss grains with sizes more than several times larger than the average size, which can be considered potential nuclei, sometimes ended as imperfections. These experimental data strongly suggest a contribution of selective growth. On the other hand, apparent experimental evidence of oriented nucleation of Goss grains has also been presented [27–29]. Dunn and Koh [30] proposed an ‘oriented nucleation growth selectivity’ theory as a compromise, and this explanation seems the most probable.

### 2.5. Inheritance of the Goss texture from hot band to secondary recrystallization

According to the experimental results described above, the subsurface layer containing Goss grains should play an important role in inducing secondary recrystallization. Böttcher and Lücke [31] found that deterioration of the Goss texture sharpness only occurred if a critical surface layer was removed from both sides of the sheet and that this phenomenon was independent of the processing stage in which removal was performed. This result suggested a kind of ‘texture inheritance’ due to a texture profile being present in a hot-rolled strip with a strong Goss texture at its surface layer. This was also supported by the observation that secondary recrystallization occurred incompletely during secondary recrystallization when the subsurface layer containing the Goss texture was removed, as reported by Mishra et al. [32]. Therefore, the inheritance of Goss grains throughout the cold rolling process seems to be closely related to the mechanism of selective growth in the secondary recrystallization of Fe-3%Si steel.

**Figure 13.**
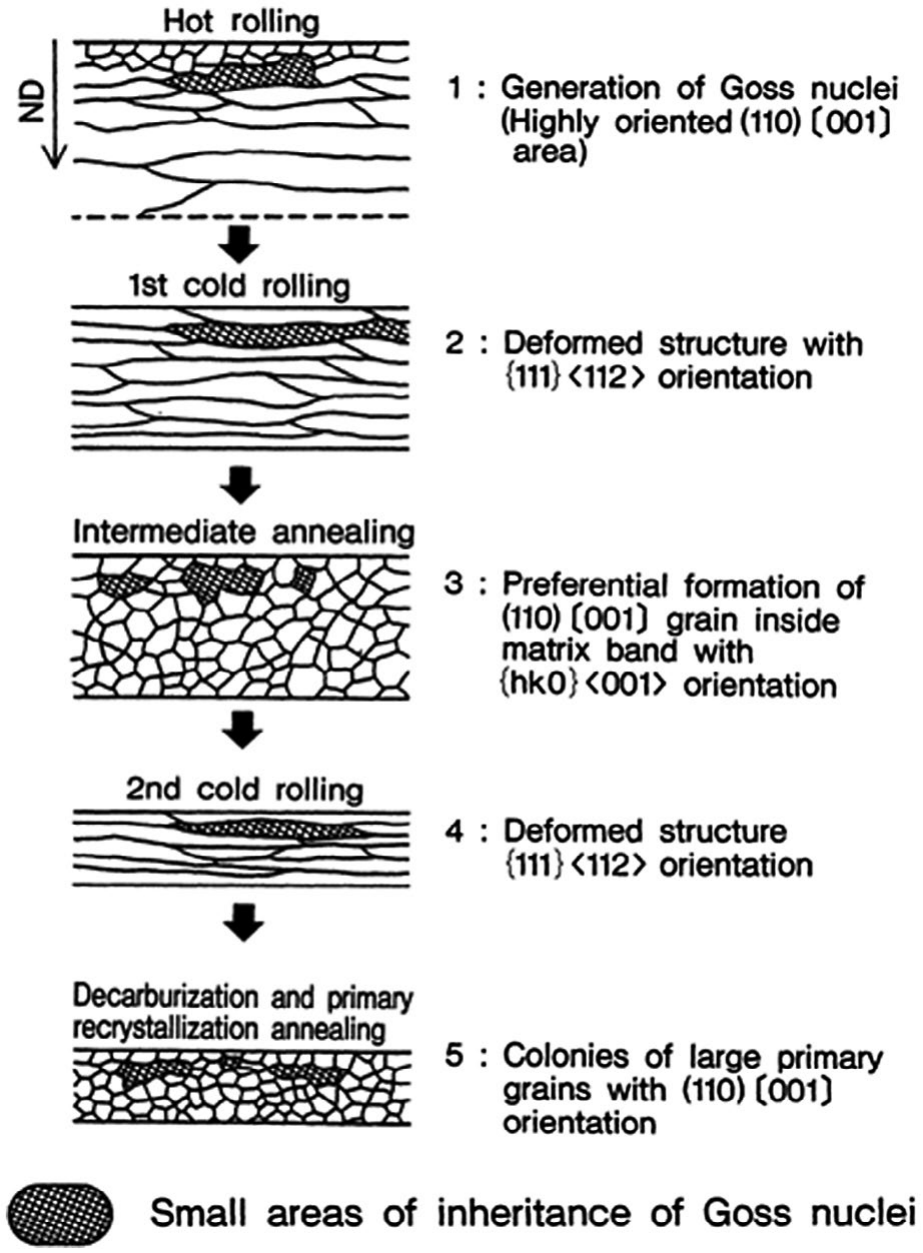
Schematic of mechanism of inheritance by structure memory for original hot-rolled silicon steel sheet up to primary recrystallization annealing [26].

A schematic of the inheritance of the Goss texture from the hot band stage is presented in Figure 13 [26].

Shimizu et al. [33] and Inokuti et al. [34] pointed out that a strong Goss texture formed in the subsurface layer and α-fiber texture RD//<110> formed in the center layer of hot-rolled silicon steel. Formation of texture gradients in hot-rolled silicon steel sheets has been the subject of a number of research studies [7,35].

The mechanisms of surface texture formation have mainly been discussed in terms of lubrication in rolling. Hashimoto [36] theoretically proved the stability of the Goss orientation under shear stress on the basis of the crystal rotation caused by slip activation.

Haratani et al. [37] and Murakami et al. [38] believed that secondary recrystallization originated from recrystallization from shear bands formed in the cold rolling process. Dorner et al. [39] studied the heritage of Goss grains during cold rolling.

Two types of Goss-oriented regions were discernable in material subjected to 89% cold rolling reduction, and it appeared that these two types of Goss regions have different origins. The Goss grains that were found aligned in shear bands formed during straining, and a second type of Goss region was found between microbands where the initial Goss orientation was retained. However, from the experimental data showing that removal of the surface layers deteriorates the Goss texture sharpness, the Goss texture which forms during hot rolling seems more important than that which forms from shear bands.

### 2.6. Effect of grain size and local texture

Selection of Goss grains from primary recrystallized grains during final annealing was treated by Dunn 60 years ago [40].

**Figure 14.**
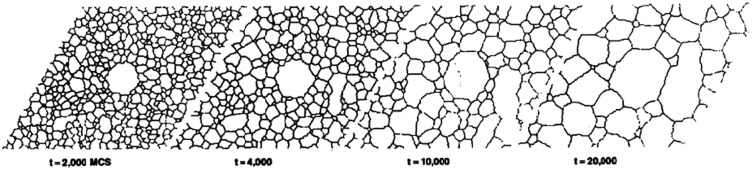
Growth of a large grain in otherwise normal grain growth microstructure. The large grain was introduced as a circular grain with the initial size of R0=5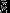 [42]. Over-lined 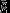 stands for average grain size.

Later, the roles of the size effect, boundary energy and mobility were investigated by theoretical [41] and computational analyses [42,43]. Srolovitz et al. showed that a very large grain will always grow more slowly than a grain of average size and will eventually rejoin the normal size distribution, as shown in Figure 14. Therefore, abnormal grain growth can only occur when normal grain growth is inhibited. In conclusion, unless the abnormally growing grain enjoys some growth advantage other than the size of its neighbors, secondary recrystallization will not be realized.

Furthermore, experimental results do not support the initial size advantage of Goss grains. In the case of high-permeability steel produced by one-stage cold rolling, Goss grains did not form colonies, nor did they have a size advantage [44–46].

Chen et al. [46] investigated the topological aspect of the primary recrystallization texture and found no tendency of grains closer to the Goss orientation to be larger. Rather, they found that the scattering of angular deviation with respect to the Goss orientation was similar over a large range of grain sizes, and this was also true when the number of next neighbors of a grain rather than its grain size was investigated. However, one single grain was found that was close to the Goss orientation and had a high number of next neighbors, and that grain might therefore act as a nucleus for secondary recrystallization.

In a Monte-Carlo simulation treating 1500 grains, Hayakawa and Szpunar [47] showed that secondary recrystallization nuclei which had a larger size were surrounded by small grains with mobile boundaries.

### 2.7. Coincidence site lattice boundary model

The idea of oriented growth has been most frequently favored as the mechanism of Goss texture formation, and several models have been proposed.

The first such model [48] suggested that coincidence site lattice (CSL) boundaries were responsible for the secondary recrystallization of Goss grains. Kronberg and Wilson [49] firstly proposed the concept of a CSL in describing the structure of the grain boundary, and its importance was indicated in their early study of secondary recrystallization in copper. Aust and Rutter [50] further demonstrated that not all grain boundaries were created equal and that the migration behavior of a particular boundary was strongly influenced by the presence of solutes. For intermediate impurities in Zn, the strong interaction of impurities and the grain boundary for mobility was found to strongly depend on the rotation angle distinguishing special and non-special boundaries [51]. The common understanding of the orientation dependence of mobility is a segregation effect: highly ordered low-Σ coincidence boundaries segregate less and, therefore, move faster than random boundaries.

**Figure 15.**
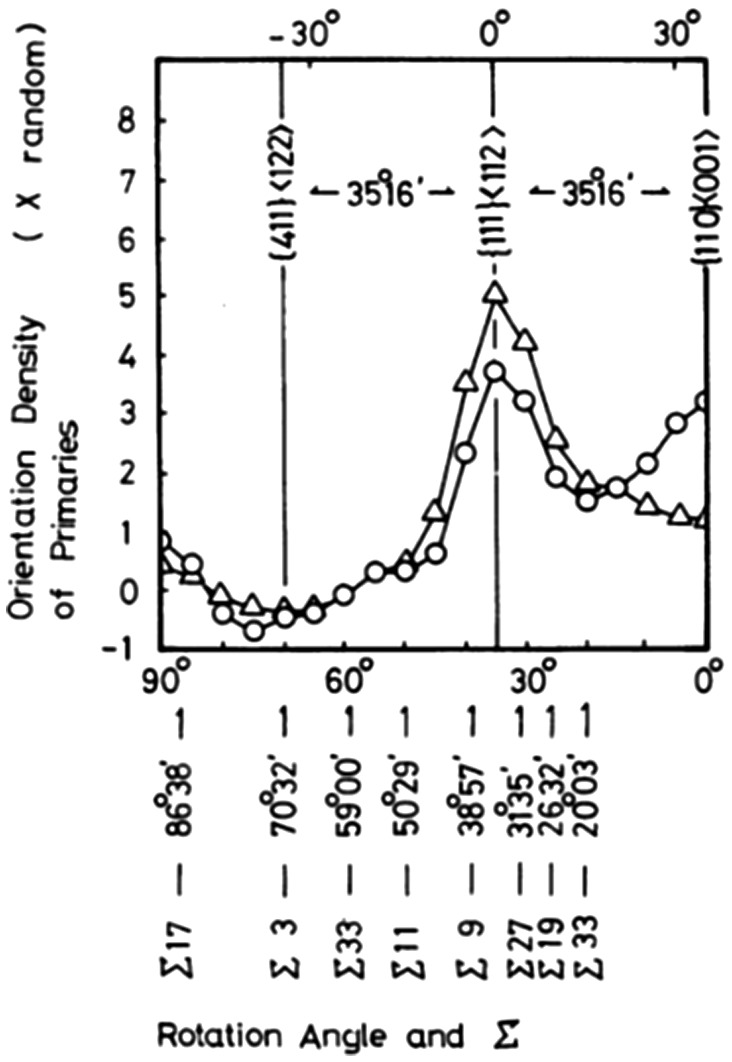
Relation between orientation density of primary recrystallization texture with TD <110> axis rotation from Goss orientation, referring to distribution of coincidence boundaries. Circles and triangles show the orientation density of the primary recrystallization texture of the cold rolling directions of 0° and 90°, respectively [52].

**Figure 16.**
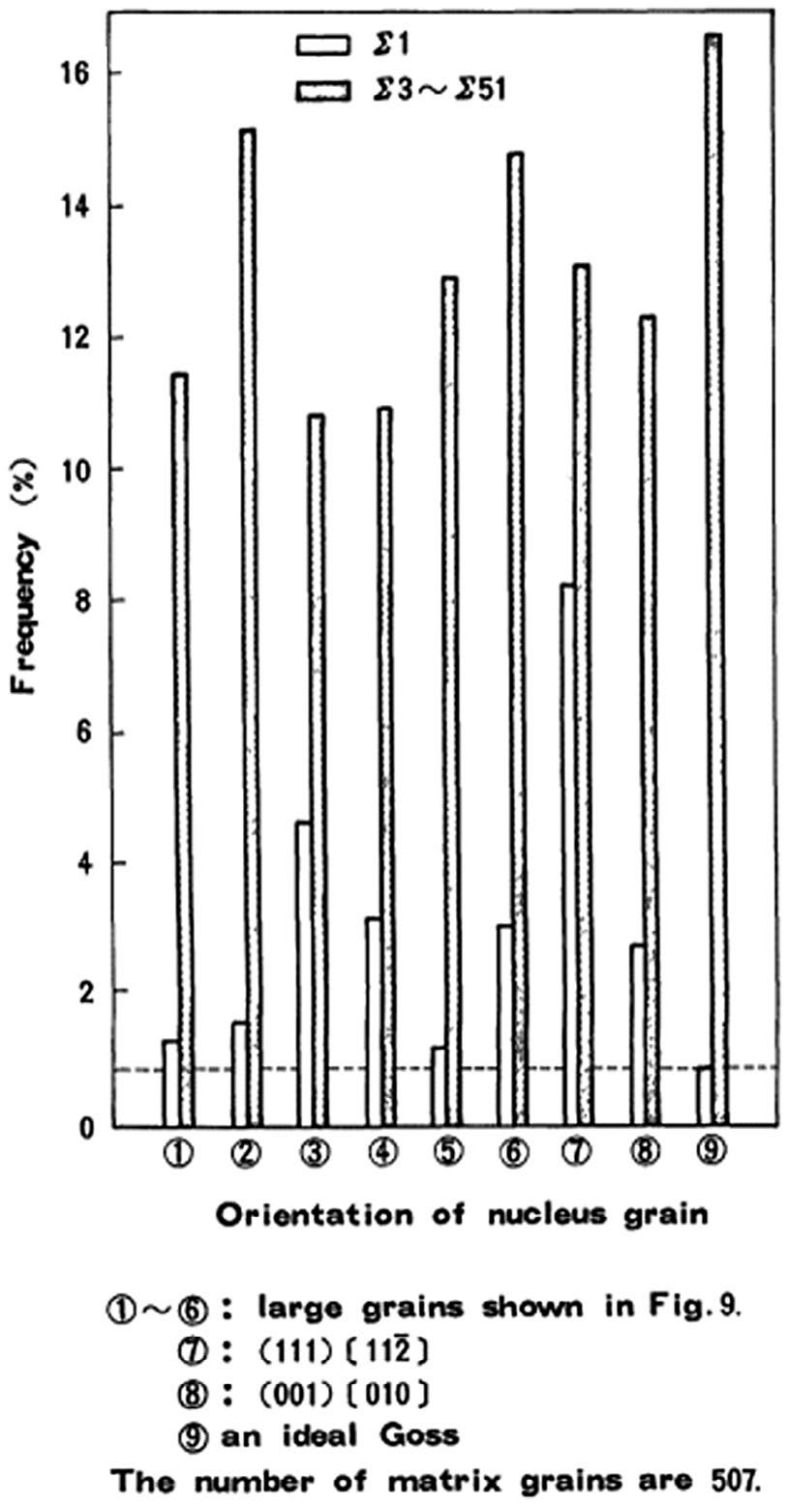
Frequency of Σ1 boundary and coincidence boundaries in primary recrystallized matrix corresponding to 6 extraordinary large grains and ideal Goss orientation and two major orientations ({111}<112>, {100}<001>) (reproduced with permission from [44] © 1988 The Japan Institute of Metals and Materials).

**Figure 17.**
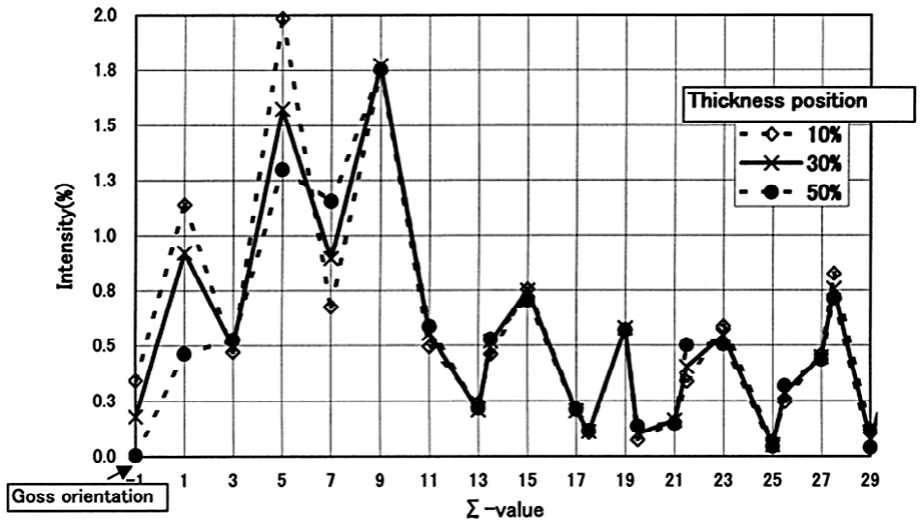
Relation between Σ value and its intensity to Goss orientation in primary recrystallization texture for different locations across the sample thickness (reproduced with permission from [53] © 2004 The Iron and Steel Institute of Japan).

Shinozaki et al. [52] compared the orientation relationship between the secondary recrystallization and primary recrystallization textures. The orientation relationship could be characterized by rotation of 35° about the common <110> axis from the Goss orientation. The grain boundary with this rotation angle was suggested to have some coincidence relationship, as shown in Figure 15. Among {110}-oriented grains, Harase and Shimizu [44] found that Goss-oriented grains had the highest frequency of CSL boundaries around them, as shown in Figure 16. Figure 17 shows the relation between the Σ value and its intensity and the Goss orientation in the primary recrystallization texture through the thickness according to Kumano et al. [53]. The intensities of Σ9 and Σ5 for the Goss orientation were stronger than those of other Σ values at each position. The main basis of the CSL boundary model has been described by Ushigami et al. [54].

### 2.8. SH method

**Figure 18.**
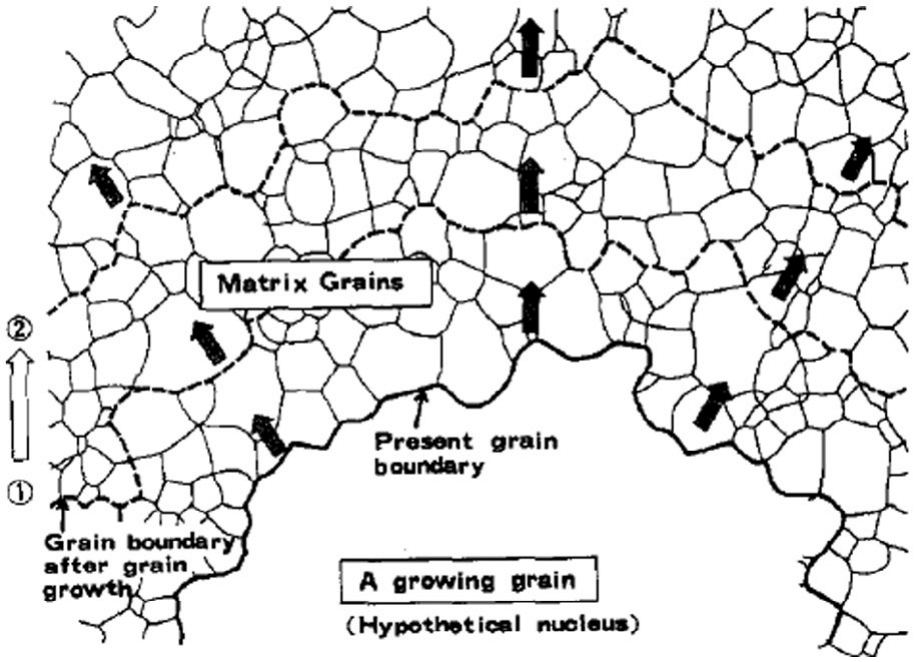
Schematic of a growing grain (hypothetical nucleus) advancing into the stable matrix and forming new grain boundaries in the process [55].

In investigating grain growth processes, it is necessary to know the characteristic of the grain boundary between a growing grain (a nucleus) and the matrix grains. In this connection, an analytical technique called the SH method (simulation by hypothetical nucleus) was developed by Shimizu et al. [55]. The SH method involves calculating the frequency of CSL grain boundaries that are likely to form with respect to a growing grain (hypothetical nucleus) from the primary matrix, to further elucidate the mechanism of secondary recrystallization in Fe-3%Si alloys. The concept is that, in the investigation of the grain growth process, in addition to the boundary relationship between grains that are in mutual contact prior to the onset of secondary recrystallization, the potential relationship when a growing grain comes into contact with other matrix grains must also be considered. This method is illustrated schematically in Figure 18 [55].

In the SH method, the orientations of several hundred grains in the matrix are measured by electron back scattering diffraction (EBSD) or obtained by calculation from the orientation distribution function (ODF). The orientation relationships between these grains and a specified nucleus orientation are then calculated. The specified orientation can be arbitrary but is usually chosen to be one of those observed to exist in the final texture after grain growth. The relationship between the frequency distribution of coincidence orientations found in this manner and the texture evolved by grain growth is then analyzed.

**Figure 19.**
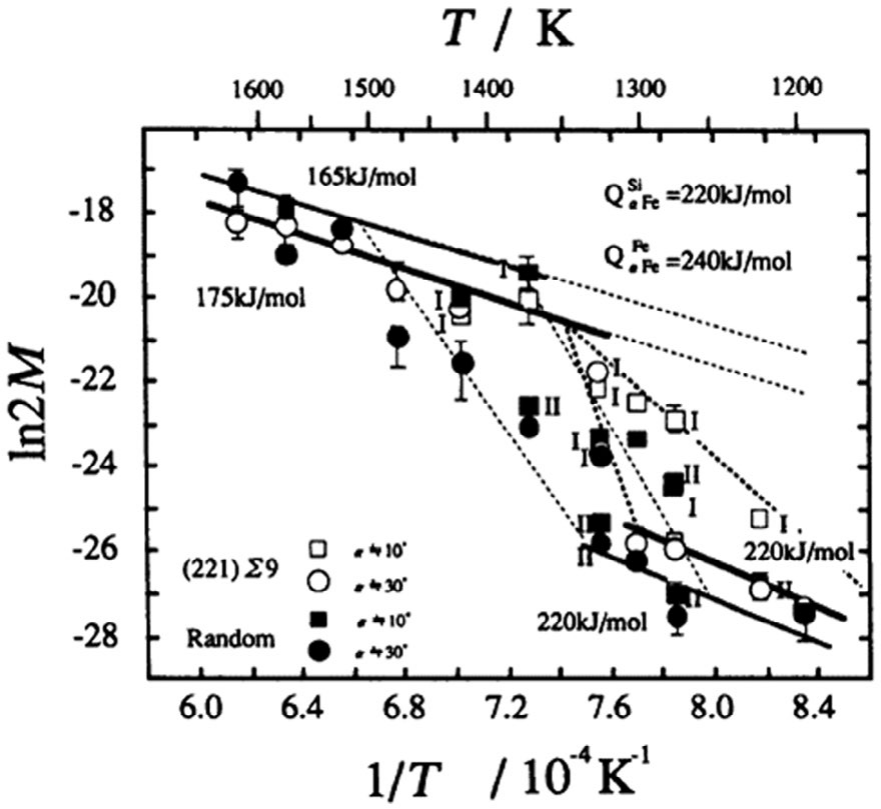
Arrhenius plots of grain boundary mobility M (reproduced with permission from [56] © 1996 The Iron and Steel Institute of Japan).

Statistically, Goss grains in a typical primary recrystallization texture have a higher probability of forming low CSL boundaries than other orientations. In a bi-crystal experiment, Nakashima et al. [56] observed that CSL boundaries migrate faster than general boundaries at temperatures between 1250 K and 1350 K. Those results are shown in Figure 19. Lin et al. [57] claimed that these CSL boundaries had higher mobility than others. However, in this context, it should be noted that the CSL boundary model has no physical meaning as such, but is a geometrical concept from which possible physical mechanisms such as grain boundary dislocations or the solubility of foreign atoms can be derived.

According to the CSL boundary model, Goss grains have the highest probability of forming CSL boundaries with neighboring grains in the primary matrix. In final batch annealing, the pinning force of the inhibitors at these boundaries will be weaker than that at the random boundaries. Therefore, these boundaries will migrate earlier, allowing Goss grains to grow at a lower temperature than other orientations or, in other words, with less dissolution of the inhibitor particles. Considering this model, some studies have suggested that Σ9 and Σ5 boundaries play an important role in the growth selection of Goss grains in GO steels [54].

### 2.9. High-energy boundary model

**Figure 20.**
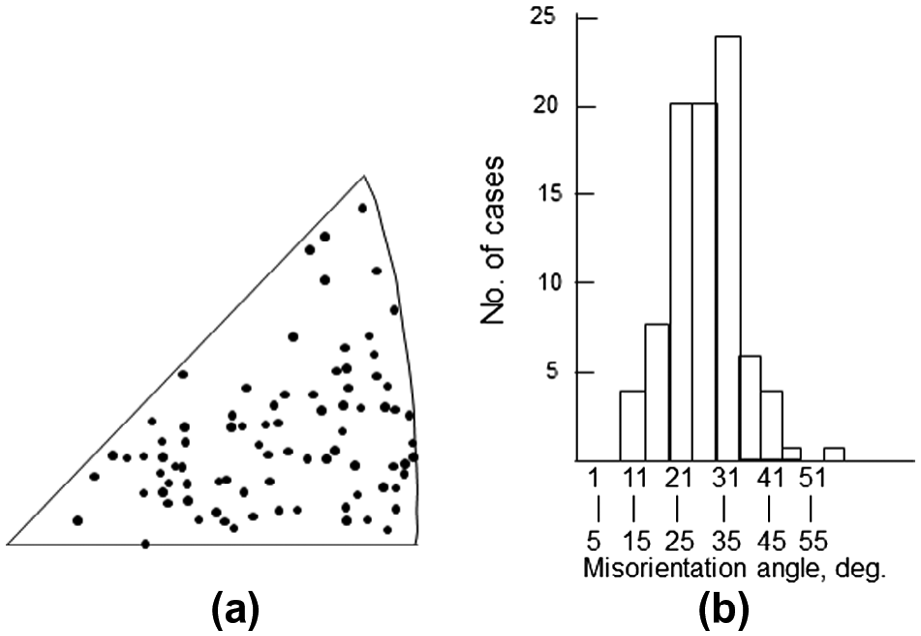
Misorientation between growing grain and major texture components of consumed matrix, (a) misorientation axis and (b) misorientation angle [58].

**Figure 21.**
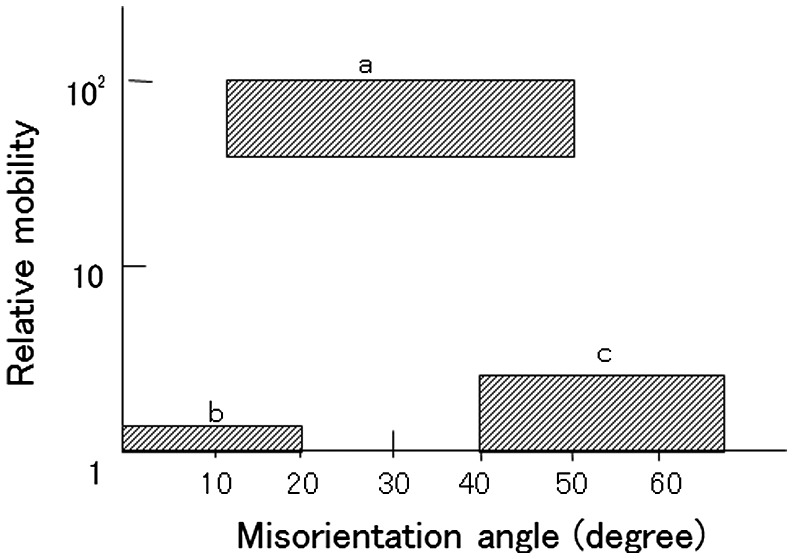
Grain boundary mobility dependence on misorientation angle suggested by Titorov [59].

Many early works concerning grain boundary mobility were published by Russian scientists. Titorov [58,59] found that the misorientation angle between the growing grain and the major texture components of the consumed matrix is in the range of 15° to 45°, and the grain boundaries were considered to be highly mobile irrespective of the type of misorientation axis, as shown in Figure 20. Titorov assumed that boundary misorientation angles from 0° to 15° have low mobility, angles from 15° to 45° have high mobility and angles from 45° to 63° have medium mobility, as shown in Figure 21. In an analysis of the theoretical pole figure of the primary recrystallization texture of Fe-3%Si, the growth of Goss grains was predicted by using the above-mentioned mobility assumptions. Gubernatorov et al. [60] were the first to relate boundary energy to boundary mobility. The misorientation angle of 35° corresponds to the maximum energy, and boundaries with this misorientation angle have the maximum mobility, which is attributed to their porous structure.

**Figure 22.**
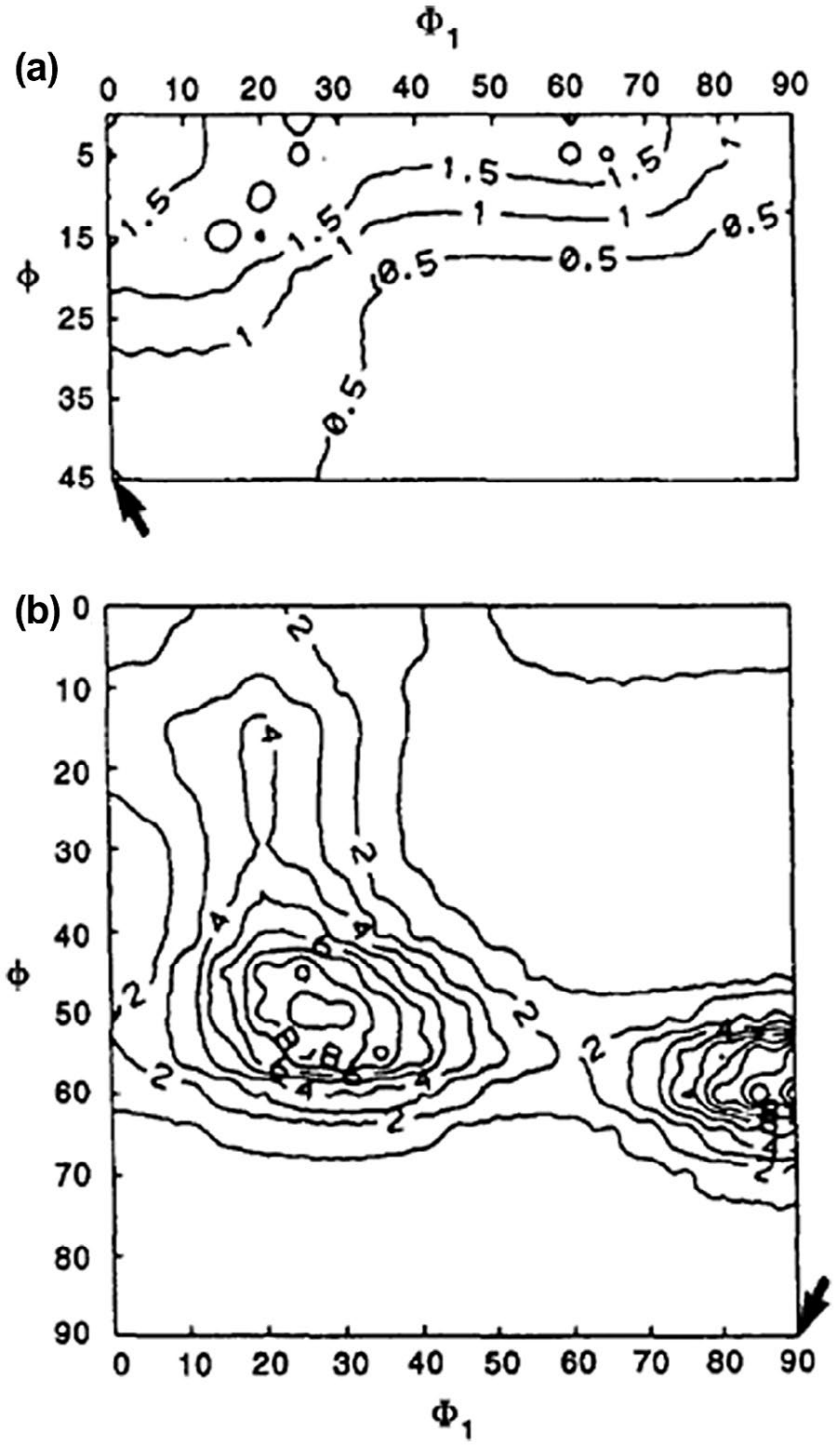
ODF of Fe-3% Si specimens after decarburizing annealing. Arrows indicate the Goss orientation: (a) cross-section Φ2=0°; (b) cross-section Φ2=45° [62].

**Figure 23.**
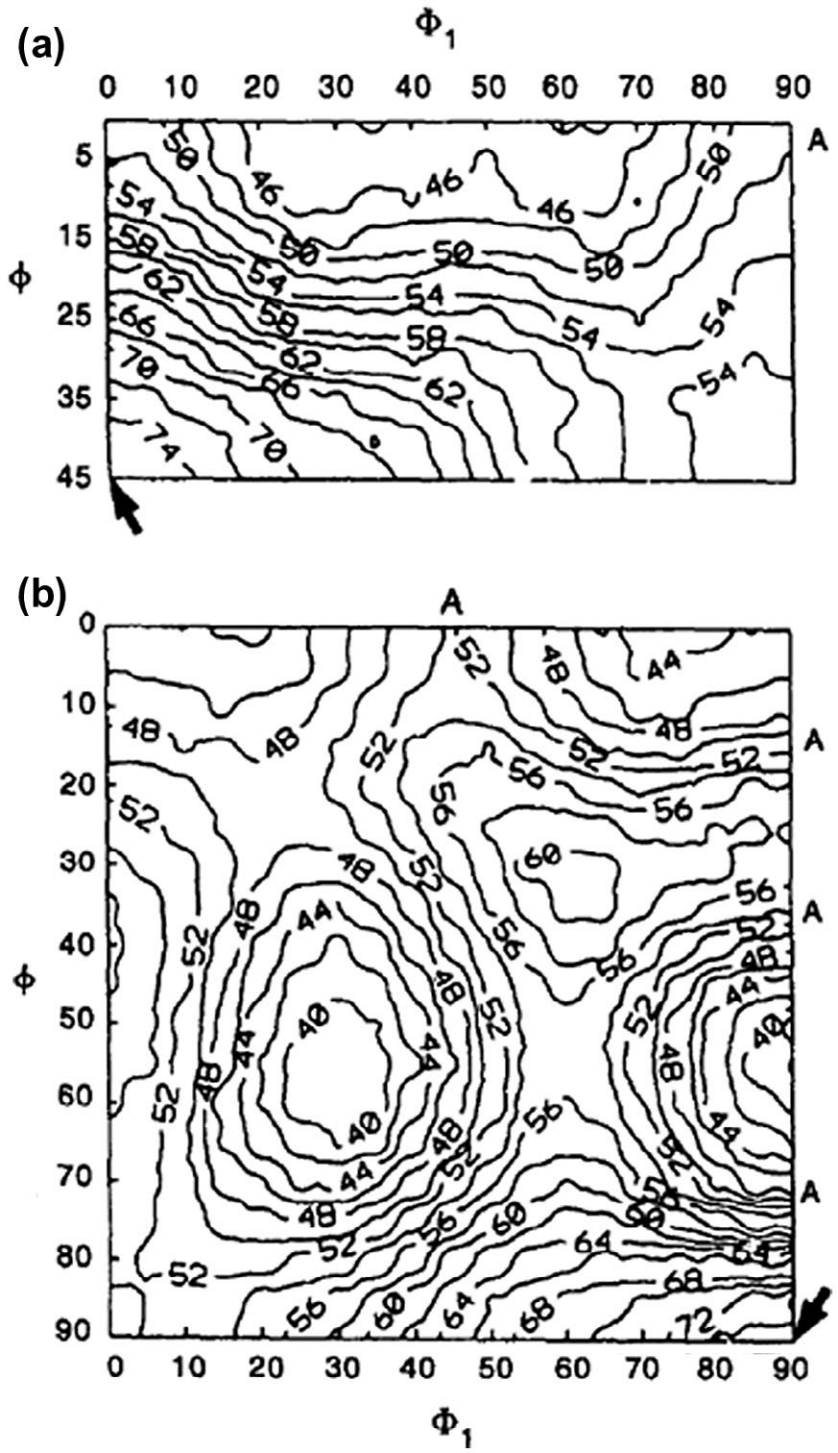
Probability (%) of boundaries having misorientation angles from 20° to 45° around grains having various orientations: (a) cross-section Φ2=0°; (b) cross-section Φ2=45° [62]. Arrows indicate the Goss orientation and the contour line of the average is indicated by ‘A’.

Hayakawa and Szpunar carried out extensive studies on the mechanism of secondary recrystallization of Goss grains. Analyses similar to the SH method by Shimizu et al. [55] were used to investigate the primary recrystallization texture of HGO steel, as shown in Figure 22 [61,62]. The conclusion was that the probability of grain boundaries having misorientation angles from 20° to 45° in the primary recrystallization texture was the highest around Goss grains among all the orientations shown in Figure 23.

**Figure 24.**
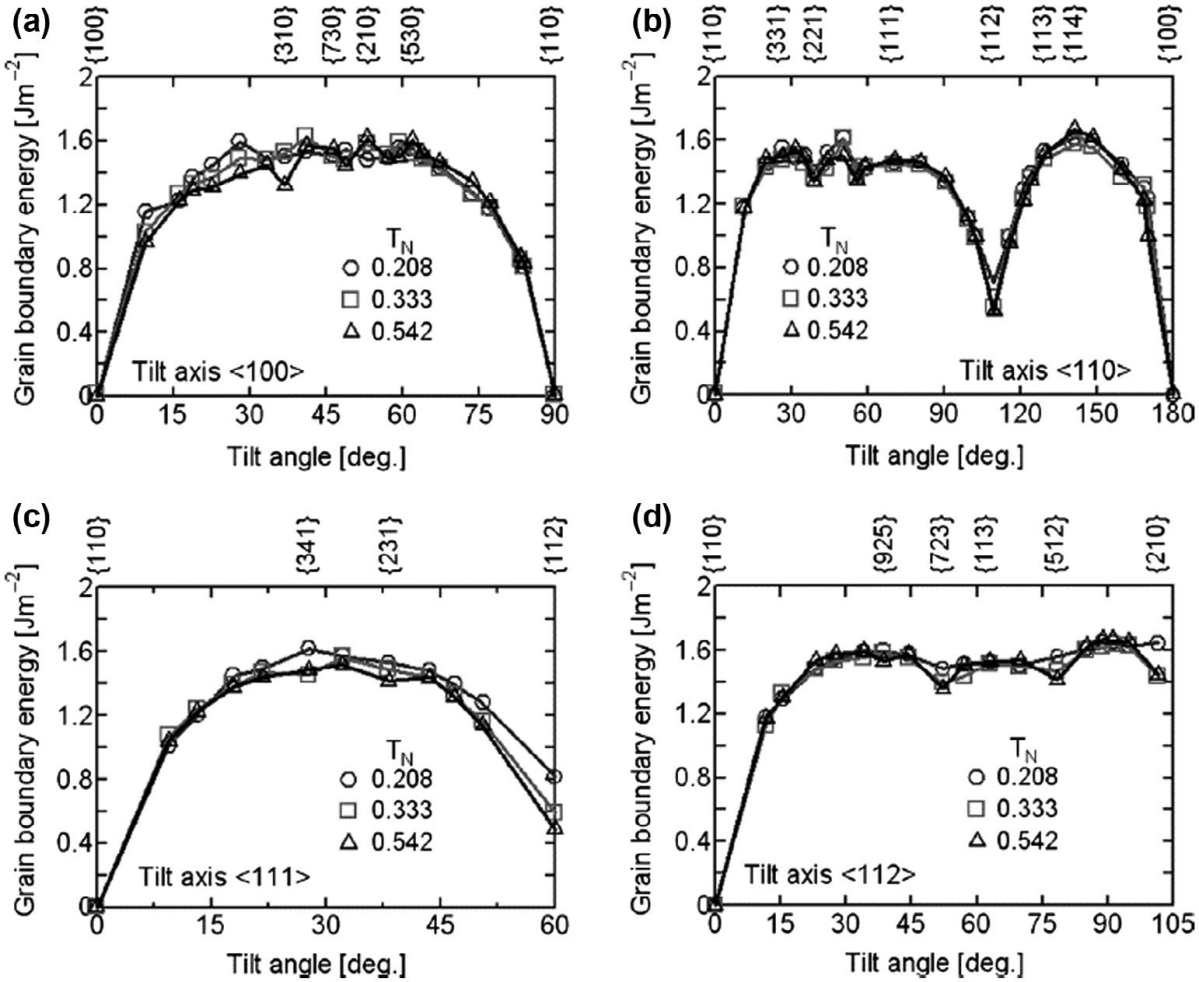
Grain boundary energy of symmetric tilt boundary in bcc phase as function of tilt angle for tilt axes of (a) <100>, (b) <110>, (c) <111> and (d) <112> (reproduced with permission from [66] © 2008 The Iron and Steel Institute of Japan).

The dependence of grain boundary energy on the grain boundary misorientation angle was recognized at an early date. Dunn and Lionetti [63] and Dunn et al. [64,65] measured the grain boundary energy as a function of the misorientation angle for (100) and (110) tilt boundaries in Fe-3%Si steel. These boundaries have misorientation angles from 20° to 45°, corresponding to high-energy (HE) boundaries. Figure 24 shows calculation results for the boundary energy in bcc phase iron by Shibuta et al. [66] using molecular dynamics.

**Figure 25.**
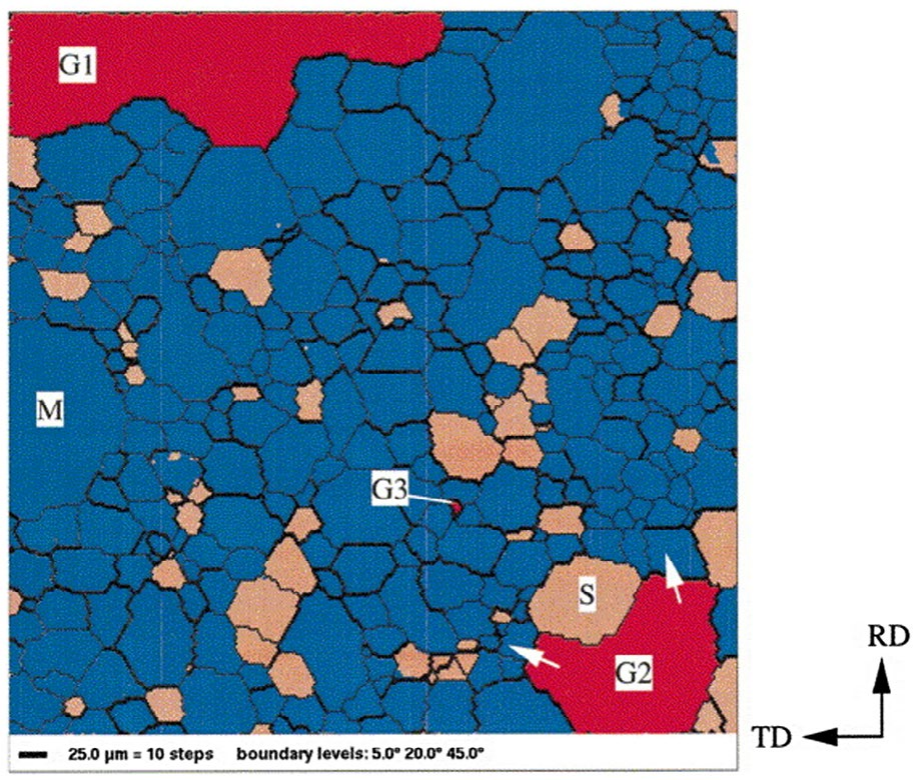
Orientation image microscopy (OIM) of specimen in secondary recrystallization. The bold black lines represent boundaries with a misorientation angle between 20° and 45°. Grains shown in red represent Goss grains with a deviation angle of less than 5° from the ideal position. Grains shown in blue represent grains with a misorientation angle of between 20° and 45° with respect to the Goss orientation [67].

**Figure 26.**
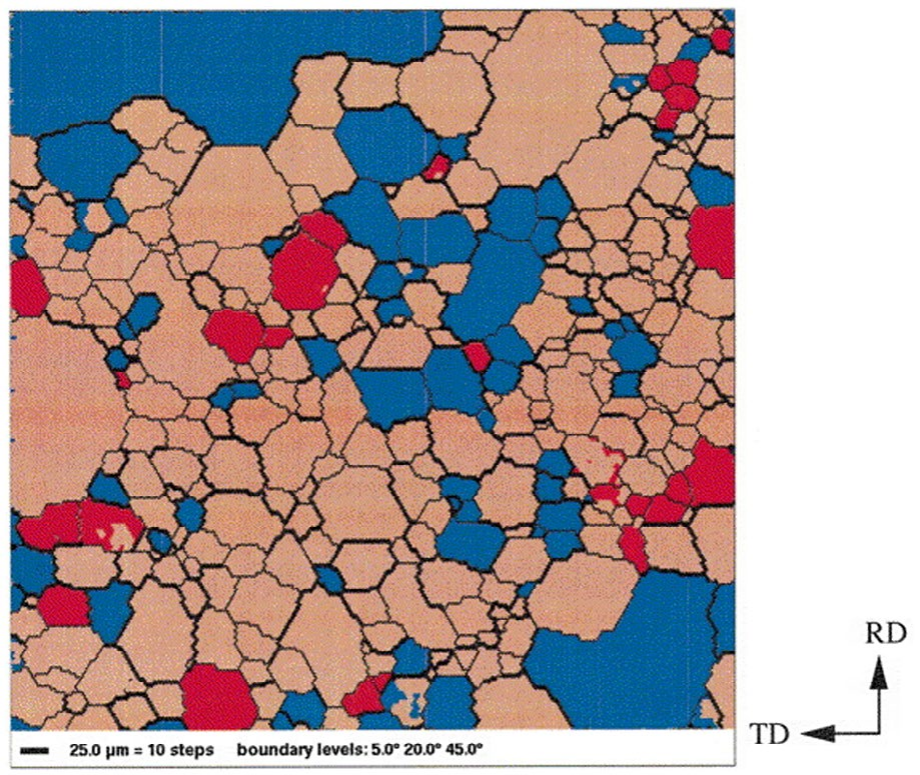
OIM image of specimen in secondary recrystallization. The bold black lines represent boundaries with a misorientation angle between 20° and 45°. Grains shown in red represent the main texture component {554}<225> having a deviation angle of less than 10° from the ideal position. Grains shown in blue represent grains with a misorientation angle of between 20° and 45° with respect to the {554}<225> orientation [67].

In the progress of secondary recrystallization, the grain orientations of HGO steel were measured by EBSD [67]. Approximately 80% of the matrix grains had misorientation angles between 20° and 45° with respect to the growing Goss grain, as shown in Figure 25. On the other hand, in Figure 26, fewer than 30% of the matrix grains had misorientation angles in this range with respect to the main texture component of the matrix {554}<225>.

**Figure 27.**
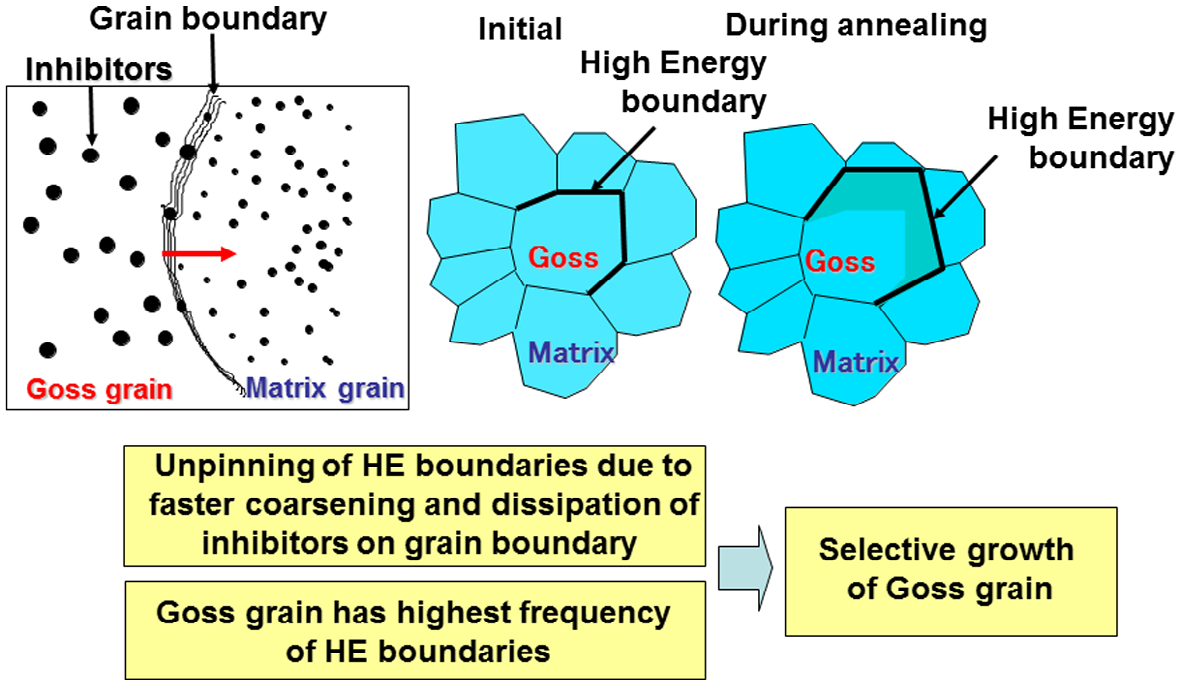
Schematic of HE boundary model of secondary recrystallization [68].

A proposed mechanism of secondary recrystallization links HE boundaries which have a disordered structure. These boundaries are often associated with a high migration rate and a high grain boundary diffusion rate, which facilitate the coarsening of inhibitors. A schematic of the HE boundary model for the mechanism of the selective growth of Goss grains is given in Figure 27 [68].

It was suggested from the HE boundary model that the primary recrystallization texture plays a more important role than inhibitors in the selection of the Goss texture during secondary recrystallization. With an appropriate texture and a composition which does not contain inhibitor constituents, secondary recrystallization proceeded through the effect of purification of the material [69].

Rajmohan et al. [70] treated the role of the fraction of mobile boundaries and concluded that a fraction of over 30% is needed in order to maintain secondary recrystallization. Since the fraction of CSL boundaries such as Σ5 or Σ9 is 1%–2%, which is much less than 30%, the validity of the contribution of CSL boundaries seems questionable.

### 2.10. Interaction between inhibitors and grain boundary

It is essentially important to treat the interaction between precipitates and the grain boundary. Classically, Zener treated the pinning effect of inhibitors on the grain boundary [71], and Gladmann [72] extended the pinning concept. However, their models did not consider the grain boundary character.

Further, Balandin et al. [73] investigated the changes in the state of inhibitors containing mainly AlN when secondary recrystallization grain boundaries pass through them.

**Figure 28.**
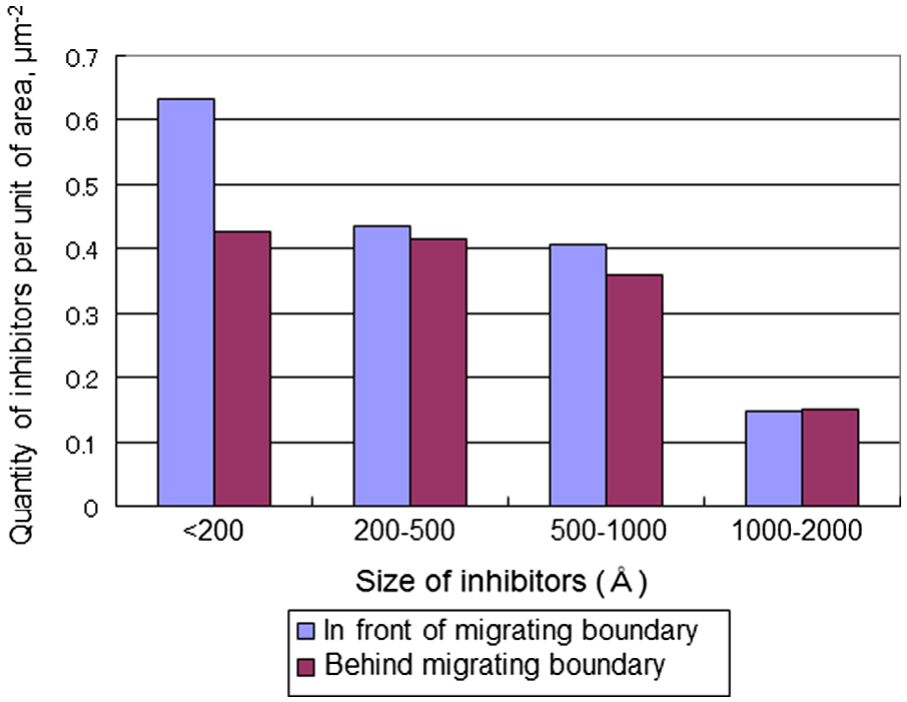
Density of inhibitors in front of and behind migrating boundary after annealing for 20 min at 930 °C [73].

The state of the inhibitors in the remaining matrix grains and large grains was investigated. The results of observation by Balandin et al. are illustrated in Figure 28. As there was a very large decrease (44%) in the number of small inhibitors <200 Å behind a moving boundary, Balandin et al. proposed a relationship involving the interaction of inhibitors with migrating grain boundaries. A similar observation was made by Gol’dshteyn et al. [74], showing that an increase in the rate of grain boundary migration increases the coalescence rate of inhibitors. The intensification of inhibitor coalescence processes during accelerated grain boundary migration was explained in terms of the higher diffusion solubility on grain boundaries.

**Figure 29.**
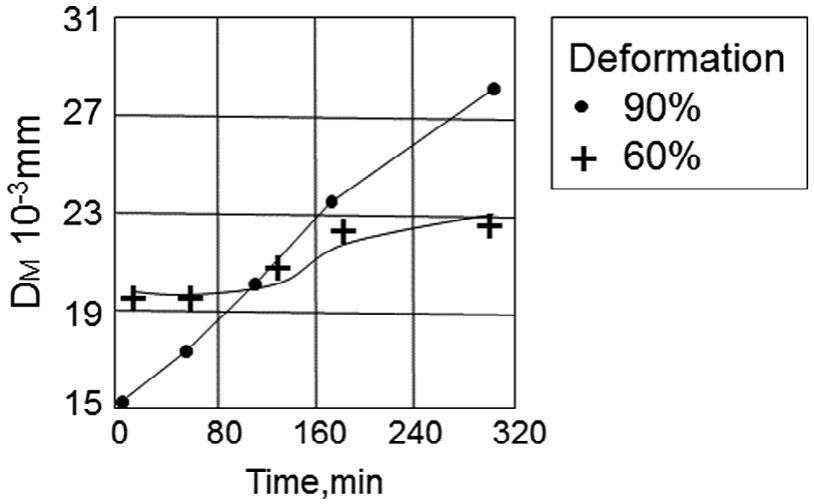
Variation in mean size of matrix grains during annealing of Fe-3%Si alloy containing AlN inhibitors at 880 °C in specimens deformed by 60% and 90% [73].

In an experiment by Balandin [73], specimens of nitrogen-containing inhibitors were cold-rolled with a reduction of 60% or 90% and then given isothermal treatment at 880 °C. In a specimen deformed by 90%, the grains became finer during primary recrystallization. The initial driving force of grain growth was higher in the specimen deformed by 90%. However, the grain continued to grow rapidly even after it had reached the size in the specimen deformed by 60%, as shown in Figure 29. Eventually, the grain size of the specimen deformed by 90% was much larger than that of the specimen deformed by 60%. Secondary recrystallization was not able to develop during high-temperature annealing in the specimen deformed by 90%, whereas the specimen deformed by 60% achieved compete development of secondary recrystallization.

Thus, if the pinning force due to inhibitors is not sufficient to stabilize the structure completely after recrystallization, the migrating boundaries may further reduce the pinning force because of the more rapid coalescence of precipitates. Thus, the interaction of dispersed particles with migrating boundaries seems to be a decisive factor in growth.

Guo and Mao [75] reported on the inhibitor distribution, showing that the selective growth of Goss grains resulted in a higher fraction of high-angle boundaries ranging from 25° to 40° than the matrix grains. On the other hand, the fraction of low-angle boundaries (<15°) surrounding matrix grains was higher than that of the grains surrounding the Goss grain, which could have a certain pinning effect on the growth of non-Goss grains. Some Goss grains had higher internal inhibitor intensities than their neighboring grains, even though they did not have a size advantage before secondary recrystallization. Grain boundary migration would meet higher resistance on moving toward the side with the higher particle density. This means that it is hard for larger grains to consume Goss grains, but rather easy for the Goss grains to engulf small neighboring grains.

### 2.11. Modelling of inhibitor growth on grain boundary

Sokolov [76] treated the coarsening of inhibitors theoretically, combining diffusion inside grains and diffusion along grain boundaries. Greenwood [77] derived a formula for coalescence of inhibitors inside grains. Ardell [78] described the growth of inhibitors as a result of diffusion along grain boundaries. Sokolov, in a first approximation, assumed the concentration of an impurity which was in equilibrium with the second phase in the region of a boundary to be proportional to the energy of that boundary. The variation in boundary energy with the misorientation angle was described by the formula of Read and Shockley [79,80].

**Figure 30.**
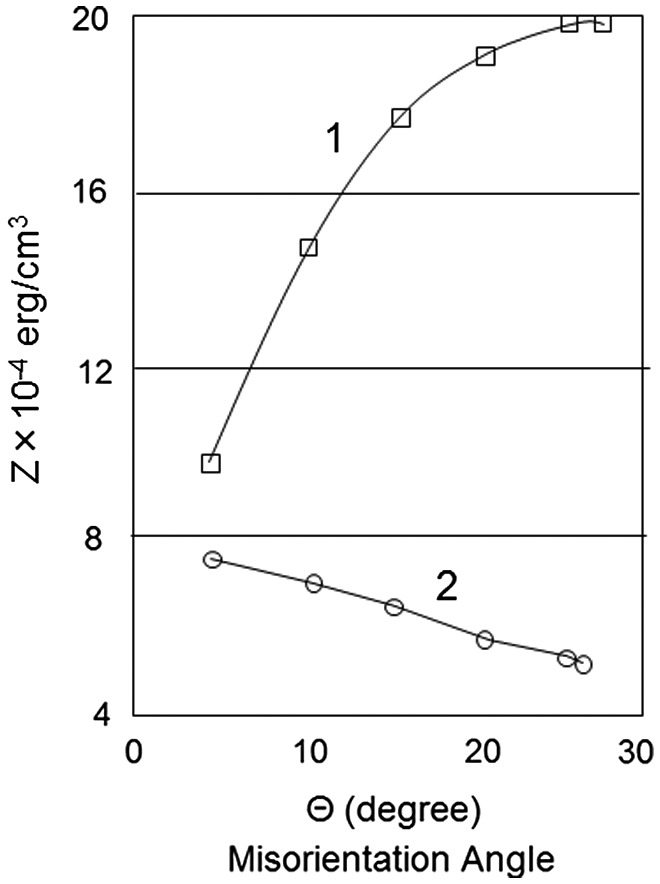
Theoretical dependence of Zener factor on misorientation angle θ at 800 °C. 1: Without allowing for influence of grain boundaries on coalescence of MnS, 2: allowing for influence of grain boundaries on coalescence [76].

A quantitative assessment of the process of coalescence was made for an alloy of iron with Fe-3%Si and containing dispersed inhibitors of manganese sulfide. The calculated result of the pinning force of the inhibitor described by the Zener term is given in Figure 30. If the influence of grain boundaries on coalescence is allowed, the pinning force diminishes as the misorientation angle increases. Otherwise, however, the pinning force increases as the misorientation angle increases in proportion to the impurity content.

Sokolov concluded as follows on the basis of these calculation results. Rather than the low-angle grain boundaries, it is the high-angle grain boundaries which have higher energy and which free themselves sooner from the pinning force of inhibitors. As this process continues to develop, the grains with the highest boundary mobility are selected. Sokolov named this the mechanism of ‘spontaneous detachment’. Interestingly, the conclusion is in good agreement with the experimental results obtained by Nakae and Tagashira [22,23].

Rajmohan and Szpunar [81] used a three-dimensional Monte-Carlo computer model to study the abnormal grain growth of Goss grains in the presence of coarsening MnS inhibitors. The calculations showed that HE boundaries were released at the early stages of the simulation. Their results also demonstrated that the intensities of Goss grains and the main texture components increased in the early stages of grain growth. In the next stage, that is, annealing, the intensity of the Goss texture increased at the expense of the main texture component, and the growth of the largest grain reached a steady-state rate.

### 2.12. Solid-state wetting model

**Figure 31.**
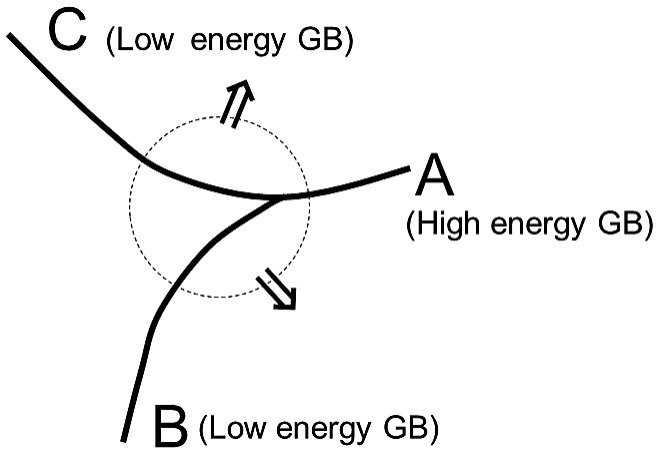
Schematic showing a high-energy grain boundary A penetrated by two low-energy grain boundaries B and C, where the energetic relation, γA > γB + γC, is satisfied (reproduced with permission from [82] © 2004 AIP Publishing LLC).

The solid-state wetting theory (SSW) [82] stresses the importance of subgrain boundaries in Goss grains with low boundary energy. This type of low-energy boundary will extrude at the triple junction of primarily recrystallized grains by wetting into the high-angle grain boundary and separating two normal grains, thus leading to the growth of the Goss grains, as illustrated in Figure 31. In this model, the growth direction of the Goss grain will be along the boundaries rather than normal to the boundaries.

**Figure 32.**
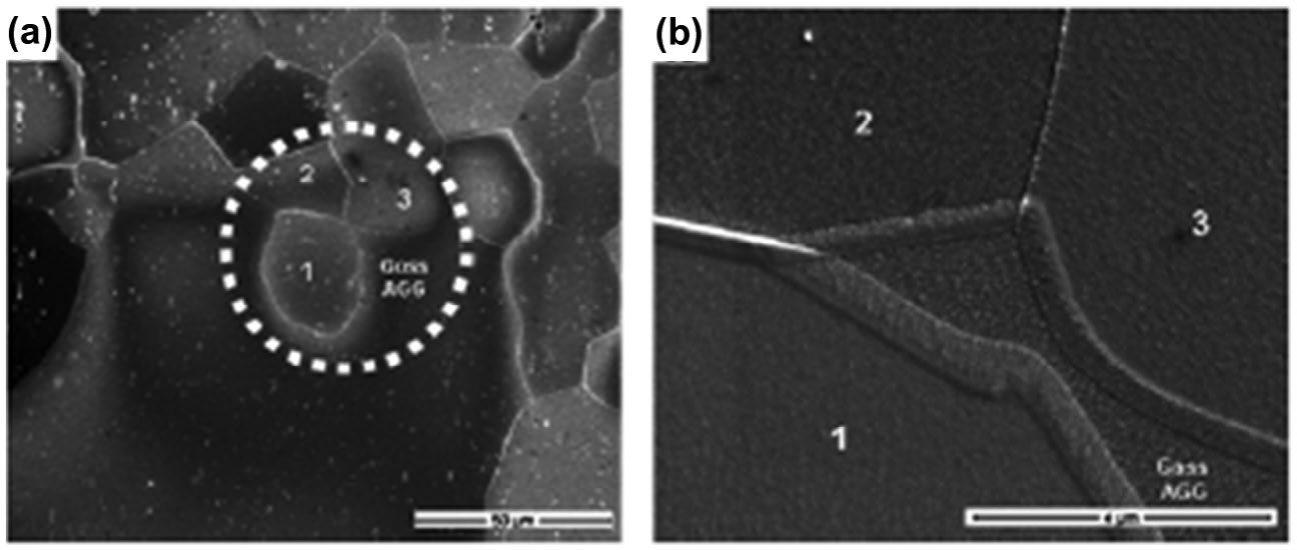
(a) SEM image of a peninsular grain ‘1’ near the front of abnormal Goss grain growth and (b) magnified image of area in white-dotted circle, showing wetting of the Goss abnormal grain growth along triple junction of grains ‘1’, ‘2’ and ‘3’ [83].

**Figure 33.**
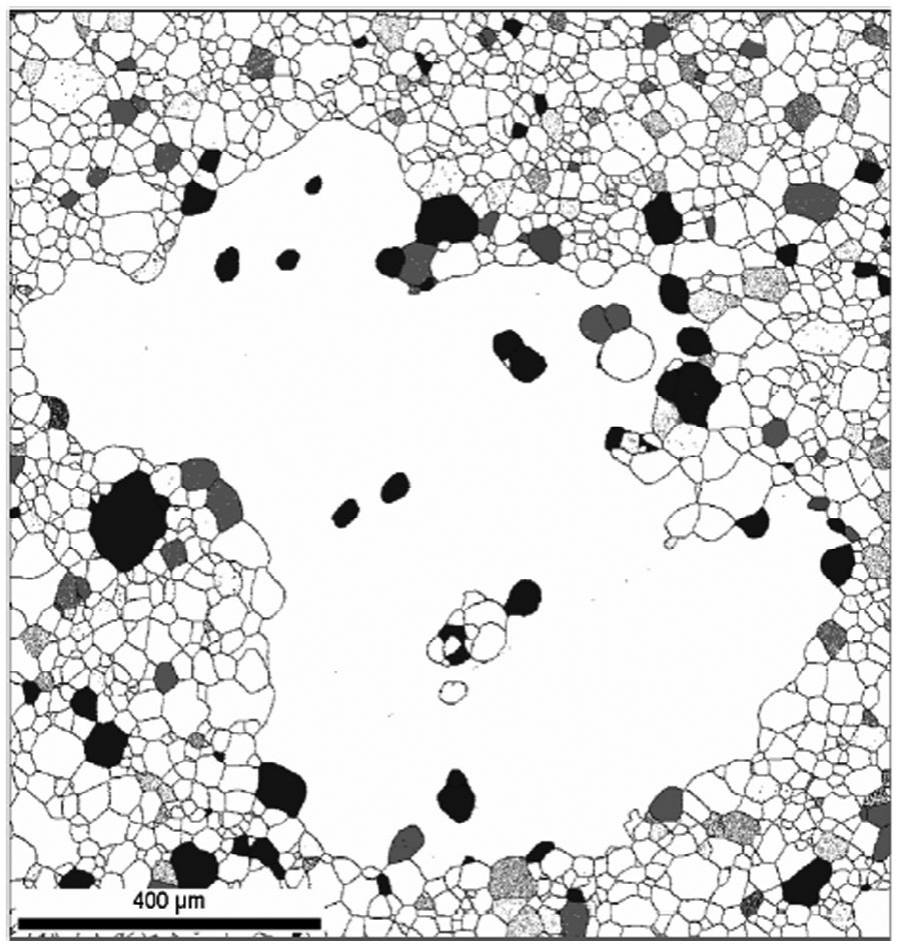
Microstructure of abnormally growing Goss grain found in Fe-3%Si steel, which contains many island and peninsular grains. Grains with misorientations below 15° with a Goss grain are shown in black. Grains with CSL relationships (Σ3~Σ37) with the Goss grain are shown in grey [84].

Ko et al. [83] provided a morphological structure by which the abnormal grain growth of Goss grains might be formed by SSW, as shown in Figure 32. Figure 33 shows a typical microstructure of an abnormally grown Goss grain in Fe-3%Si steel provided by Lee et al. [84]. Many island and peninsular grains are observed inside the Goss grain. Most island grains are black and could be considered to have relatively low energy boundaries. Messina et al. [85] and Ko et al. [86] suggested that island and peninsular grains are formed by SSW.

Lee et al. [87] and Ko et al. [88] have shown by Monte-Carlo (MC) simulations and phase-field model simulations, respectively, that a grain which contained subgrain boundaries of very low energy had an exclusive growth advantage over other grains and underwent secondary recrystallization.

The study by Messina et al. [85], which compared the mobility and energy advantages of the grain boundary in inducing secondary recrystallization, showed that the percentages of high-mobility and low-energy grain boundaries required for secondary recrystallization should be at least 50% and 20%, respectively.

### 2.13. Vacancy model

In addition to these misorientation schemes, another model of secondary recrystallization was proposed by Titorov [89]. Titorov [90] compared the normal growth rate and abnormal grain growth rate that proceeded concurrently within the same sample.

The secondary grains and matrix grains grew simultaneously, but the rate of matrix grain growth was 3 orders less than that of the secondary grains. Titorov insisted that such a 3-order difference cannot develop at the same temperature and similar driving force.

Moreover, since the experimental data showed that the migration ability between boundaries was rather close [58], it would be unreasonable to think that such a large difference between the growth rates of the secondary grains and the matrix grains could result from the difference in misorientation and structure of the boundaries.

Consequently, the difference in the kinetics between abnormal and normal grain growth is not related to either the difference in driving forces or the migration ability of the grain boundaries.

Titorov [90] suggested that a decrease of the inhibitor-matrix interface area occurs in the process of dissolution and coagulation of inhibitors, and showed that the energy released during the coalescence of the inhibitors due to the decrease of the area of the inhibitor-matrix boundaries was comparable or even higher than that released as a result of the decrease of the interphase surface during transformation of a fine-grain structure to a coarse-grain one in the process of secondary recrystallization.

Titorov assumed that vacancies arise in the material in the stage of dissolution of small inhibitors and diffusion of its constitutive elements toward larger inhibitors. Thus, the source of these vacancies is the volumes previously occupied by the dissolved inhibitors. Since the appearance of excess vacancies during dissolution of inhibitors is presumably a prerequisite for secondary recrystallization, Titorov introduced the idea of growth promoted by the generation of excess vacancies.

Because a grain boundary can serve as a sink for excess vacancies, there are fewer vacancies in the volume behind the moving boundary than in the volume in front of it [91]. As the diffusion permeability of the boundary increases, increasingly complete dissolution and coagulation occurs in the volumes behind the boundary. Consequently, the difference in the excess vacancy concentrations must be higher, which means that such boundaries will move faster. Thus, from the vacancy mechanism, it can be concluded that the migration of HE boundaries with higher diffusion permeability is accelerated by the dissolution of inhibitors.

## 3. Future prospects for investigation of the mechanism of secondary recrystallization

### 3.1. Criticism of proposed models

The above-mentioned three models (CSL, HE, SSW) based on boundary character distribution were criticized by Morawiec [92], who concluded that possible differences in grain boundary mobility might not be the sole reason for the abnormal grain growth of Goss grains. Using local orientation measurements and Monte-Carlo simulations, Chen et al. [93], as well as Titorov [90], pointed out that differences in mobility alone did not seem to be sufficient for the abnormal grain growth of Goss-oriented crystals. Moreover, it is also questionable whether a merely statistical link between the Goss orientation and a particular grain boundary character (CSL, HE, SSW) is able to create a texture as sharp as the observed Goss texture.

Morawiec [94] made further critical comments against the three models. He insisted that the texture evolution and sharpness of the final texture in GO silicon iron alloys can be explained if grains with low surface free energy have a high probability of growth. Yan et al. [95] discussed the possible role of surface energy. However, estimated surface energy seems insufficient for the growth, and surface oxides are usually formed, thus further reducing surface energy.

### 3.2. Future prospects

This paper has described only some of the conceivable mechanisms of secondary recrystallization of Goss grains, and many gaps remain to be filled. As ongoing models are related to the static configuration of grains or grain boundaries, dynamic observation such as using *in situ* EBSD investigation during secondary recrystallization will be useful [96]. The use of neutron diffraction or synchrotron radiation are methods for non-destructive measurement of the texture development. They will also provide bulk information on the progress of secondary recrystallization.

The vacancy model proposed by Titorov [90] appears to be a promising direction for research as vacancy is considered as an additional driving force relating to the dissolution of inhibitors. Vacancies can be detected using positron annihilation spectroscopy [97], and positron probe microanalyzer has become an efficient tool for studying the spatial distribution of vacancies [98]. Measurement of vacancy concentration during secondary recrystallization may be a solution. Based on the true secondary recrystallization mechanism, further development of GO steel is expected in future.

## Disclosure statement

No potential conflict of interest was reported by the author.
